# Quantitative CT parameters correlate with lung function in chronic obstructive pulmonary disease: A systematic review and meta-analysis

**DOI:** 10.3389/fsurg.2022.1066031

**Published:** 2023-01-04

**Authors:** Yan Wang, Limin Chai, Yuqian Chen, Jin Liu, Qingting Wang, Qianqian Zhang, Yuanjie Qiu, Danyang Li, Huan Chen, Nirui Shen, Xiangyu Shi, Jian Wang, Xinming Xie, Manxiang Li

**Affiliations:** Department of Respiratory and Critical Care Medicine, The First Affiliated Hospital of Xi’an Jiaotong University, Xi’an, China

**Keywords:** airway obstruction, systematic review, meta-analysis, quantitative CT, COPD

## Abstract

**Objective:**

This study aimed to analyze the correlation between quantitative computed tomography (CT) parameters and airflow obstruction in patients with COPD.

**Methods:**

PubMed, Embase, Cochrane and Web of Knowledge were searched by two investigators from inception to July 2022, using a combination of pertinent items to discover articles that investigated the relationship between CT measurements and lung function parameters in patients with COPD. Five reviewers independently extracted data, and evaluated it for quality and bias. The correlation coefficient was calculated, and heterogeneity was explored. The following CT measurements were extracted: percentage of lung attenuation area <−950 Hounsfield Units (HU), mean lung density, percentage of airway wall area, air trapping index, and airway wall thickness. Two airflow obstruction parameters were extracted: forced expiratory volume in the first second as a percentage of prediction (FEV_1_%pred) and FEV_1_ divided by forced expiratory volume lung capacity.

**Results:**

A total of 141 studies (25,214 participants) were identified, which 64 (6,341 participants) were suitable for our meta-analysis. Results from our analysis demonstrated that there was a significant correlation between quantitative CT parameters and lung function. The absolute pooled correlation coefficients ranged from 0.26 (95% CI, 0.18 to 0.33) to 0.70 (95% CI, 0.65 to 0.75) for inspiratory CT and 0.56 (95% CI, 0.51 to 0.60) to 0.74 (95% CI, 0.68 to 0.80) for expiratory CT.

**Conclusions:**

Results from this analysis demonstrated that quantitative CT parameters are significantly correlated with lung function in patients with COPD. With recent advances in chest CT, we can evaluate morphological features in the lungs that cannot be obtained by other clinical indices, such as pulmonary function tests. Therefore, CT can provide a quantitative method to advance the development and testing of new interventions and therapies for patients with COPD.

## Key points

(1) CT provides a quantitative and localized morphological method to study airflow limitation in COPD
(2) CT measurements have certain value for screening pre-COPD patients who have developed clinical symptoms but have not met the diagnostic criteria
(3) The combination of inspiratory and expiratory two-phase CT measurements and lung function can more accurately assess the degree of airflow limitation in patients

## Introduction

Chronic obstructive pulmonary disease (COPD) is characterized by persistent respiratory symptoms and airflow limitation (1–3). Although the majority of patients followed a path of disease progression in which the severity of COPD tracked the severity of airflow limitation, the conventional method (such as pulmonary function tests, PFTs) fail to provide – information about regional pulmonary dysfunction (4). Studies have shown that the use of the fixed forced expiratory volume in the first second/forced vital capacity (FEV_1_/FVC) ratio to define airflow limitation may result in more frequent diagnoses of COPD in the elderly, and less frequent diagnoses in adults < 45 years of age (especially in patients with mild COPD), as compared with the use of the low limit of normal (LLN) values for FEV_1_/FVC (5, 6). The research results suggested that using lung function test in diagnosis and evaluation of COPD severity is controversial (7–10).

With the rapid development of CT imaging technology, quantitative CT can help physicians to quantify and localize the relative volumes of emphysema and gas trapping in patients with COPD by using low attenuation areas (11). In addition, the COPDGene® Study evaluating the accuracy of the GOLD stages in diagnosing airflow obstruction demonstrated that PFTs do not reflect the severity of quantitative CT parameters accurately (12). Trough objective quantitative evaluation of pulmonary emphysema and airways disease, CT may help achieve clinically meaningful phenotyping. Quantitative imaging has provided repeatable and unbised estimates of the severity and distribution of lung pathology. Lung volume reduction (LVR) and Endoscopic lung volume reduction (ELVR) are the current treatments for severe emphysema in addition to lung transplantation (11). Nowdays, Quantitative CT with its increasing possibilities has become a viable tool to provide detailed information on the distribution and heterogeneity of emphysema. This structural and functional information provides support for thoracic surgeons and interventional pulmonologists to select patients and optimize LVR procedures, as well as for the development of new endobronchial therapies to further improve outcomes in patients with lung volume reduction (11–13). Thus, quantitative CT may be used as a supplementary method to assess the degree and location of airflow limitation in patients with COPD.

Recently, it has been confirmed that the quantitative CT parameters of patients with COPD are related to airflow limitation. However, the results of these confounding factors such as the difference between the inspiratory and expiratory phases, the quantitative CT machine brand, and the level of tracheal measurement are variable and sometimes contradictory (13–16). Therefore, we conducted a systematic review and meta-analysis to analyze the correlation between quantitative computed tomography (CT) parameters and airflow obstruction in patients with chronic obstructive pulmonary disease (COPD).

## Materials and methods

### Study design

The study was designed according to the Preferred Reporting Items for Systematic Reviews and Meta-Analyses (PRISMA) statement (17).

### Data sources and searches

PubMed, Embase, Cochrane and Web of Knowledge were searched for articles published from their inception to July 2022, using a combination of pertinent items to discover articles that investigated the relationship between CT measurements and lung function parameters in patients with COPD. Language restrictions were not implemented. Generally, the literature search was conducted using three keywords, such as “chronic obstructive pulmonary disease”, “pulmonary function test”, and “CT”. Besides, the Boolean operator “AND” was used in these three sets of keywords, and “OR” was used within each group. The detailed search process is shown in Supplementary Table S1.

### Study selection

Each study was evaluated independently and systematically by five investigators with more than 6 years of thoracic radiology related working experiences. Articles were included in the systematic review if they met the following criteria: (1) all patients with stage 0-IV COPD were ≥18 years old, without a history of dementia and have no changes in medication or acute exacerbation within the past 6 weeks; (2) interventions included participants who had clearly described PFT, according to the guidelines of the American Thoracic Society (ATS), the European Respiratory Society (ERS), or other similar methods; (3) the relationship between quantitative CT and PFT was analyzed; (4) and the methods of the study included randomized control trial (RCTs), observational (prospective and retrospective cohort) studies, and cross-sectional studies. In addition, the exclusion criteria were as follows: (1) case reports, letters, and conference abstracts; (2) studies with outcomes from only PFT or quantitative CT; (3) studies that included participants who were included in other studies within the past 6 weeks; (4) studies that included participants with other confounding diseases (such as interstitial lung disease, *α* − 1 anti-trypsin, asthma, lung cancer, lung surgery, active pulmonary tuberculosis, etc.); (5) studies that included participants with diseases that affected adequate breathing.

Articles were included in the meta-analysis if (1) the study included a comparable proportion of GOLD 1–4 grades patients; (2) had a sample size ≥ 20 [20 subjects would provide a power of 0.90 when detecting a typical effect correlation coefficient (CC) of 0.60]; (3) provided the percentage of lung attenuation area under −950 HU (%LAA-950), mean lung density (MLD), wall area percentage (WA%) in airways ≥ fifth airway generation, air trapping index (ATI), airway wall thickness (WT), and airway lumen area (AI) by volumetric multi-detector CT (MDCT); (4) CCs of lung function and quantitative CT; (5) and parameters of lung function included the predicted forced expiratory volume in the first second as percentage (FEV1%pred) and FEV1/FVC. Articles were excluded in the meta-analysis if: (1) Selection Bias; (2) Sample size <20; (3) Not MDCT volume scan; (4) No CT quantitative parameters and PFT parameters included.

### Data extraction and quality assessment

Two reviewers independently screened articles for the fulfillment of inclusion and exclusion criteria. Any disagreements or discrepancies were resolved through a consensus. A standardised extraction form was made to extract all relevant data from texts, tables, and figures of each study, including study characteristics, participant characteristics, methodology, and CCs. %LAA-950, %LAA < 950, MLD, 15 percentile point of lung density (Perc15), lung volume (LV), WA%, WT, AI and ATI were recorded in the systematic review. Seven CT measurements including %LAA-950, %LAA < −950 (between −856 and −950 HU), MLD, WA%, WT, AI, and ATI, were pooled in the meta-analysis and two PFT parameters including FEV_1_%pred and FEV_1_/FVC were extracted.

Furthermore, the Quality Assessment of Diagnostic Accuracy Studies (QUADAS) tool (18) was used to study methodological quality and potential bias. A study with a QUADAS score ≥11 points was deemed as high quality, while was considered to be of low quality.

### Data synthesis and analysis

The overall measure was the correlation coefficient (CC) between CT and PFT parameters. The Hedges-Vevea random-effects model and normality *Z*-test were used to calculate the pooled 95% confidence intervals (95% CIs). We calculated correlations between emphysema proportions, mean lung density, tube wall thickness, and measures of lung function. We also performed a subgroup analysis across different CT brands to find sources of heterogeneity. If evaluating multiple layers of bronchi, we chose the smallest bronchi. Heterogeneity was evaluated using the *I*^2^ index. Additionally, we used a random-effects model because this was better equipped to explain the heterogeneity between the studies. Subgroup analyses were used to determine the impact of individual variables and the potential sources of heterogeneity. Subgroups were based on radiation dose (low or normal dose) and breath-hold (inspiratory and expiratory). Meta-regression was performed to investigate the influence of gender, if only the male patients was reported by at least three studies. Also, the potential publication bias was assessed using Begg's test.

In addition, we divided the densitometric thresholds into 950 HU and <−950 HU (between −856 and −950 HU) and divided breath-holding procedures into inspiratory and expiratory. We also divided CT machines into GE and Siemens, Toshiba, and Philips. All statistical analyses were performed using Stata 15.0 and SPSS 20.0. Further, sensitivity analyses were conducted to assess the impact of each study on the results of the pooled study by eliminating each study. Finally, analysis trimming and filling were carried out if necessary.

## Results

### Study selection

**Figure 1:**
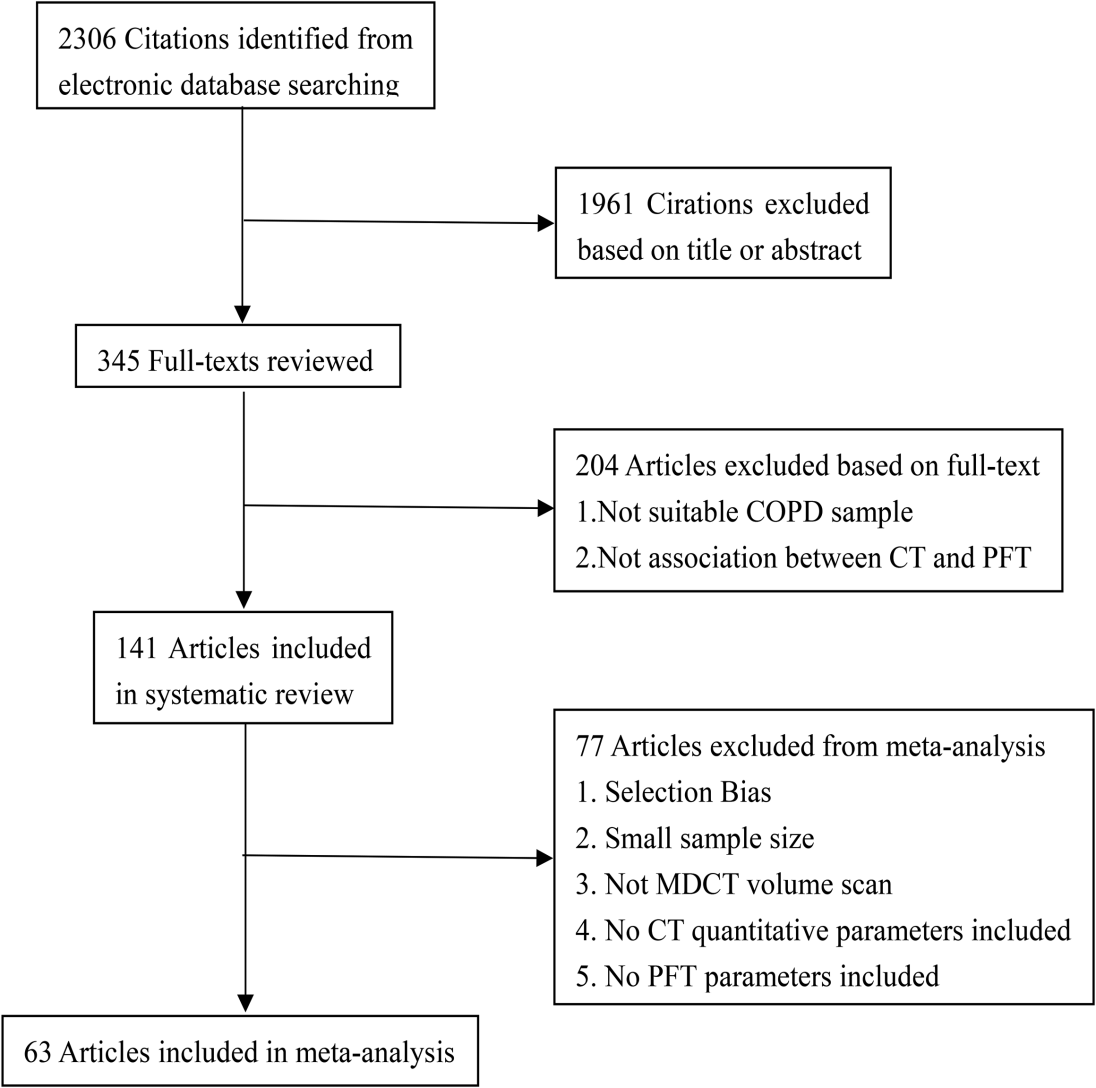
Flowchart of literature review and selection. COPD, chronic obstructive pulmonary disease; PFT, pulmonary function test; MDCT, multi-detector computed tomography.

From the electronic databases (PubMed, Embase, Cochrane and Web of Knowledge), a total of 2,306 studies were included (Figure 1), and 1,961 citations were excluded based on their titles or abstracts. After screening the text of 345 articles, 204 studies were excluded from the systematic review. The reasons for exclusion were that the studies did not include: a large enough COPD sample or there was no association between quantitative CT and PFTs. According to the exclusion criteria of the meta-analysis, 78 studies were excluded. The reasons for exclusion were as follows: (1) selection bias, (2) small sample size, (3) MDCT volume scans were not available, (4) quantitative CT parameters or PFT parameters were not included. Finally, 141 articles were used for systematic review and 63 articles were included in the meta-analysis.

### Systematic review

The systematic review included a total of 25,214 participants. The age of patients range from 20 to 90, and there were 12,252 (60.4%) men, 5,676 (30.7%) women, 2,014 (8.9%) of the participants did not specify their gender (Supplementary Tables S2, S3). This study included RCTs and cohort studies. Of these, articles 46.8% (66 articles) were from Europe, 36.1% (51 articles) were from Asia, 12.8% (18 articles) were from North America, 2.8% (4 articles) from Oceania, 1.4% (2 articles) from Africa. Further, 123 (87.2%) were written in English, 11 (7.8%) in Chinese, 5 (3.5%) in Italian, 1 (0.7%) in French, and 1 (0.7%) in Polish.

**Figure 2:**
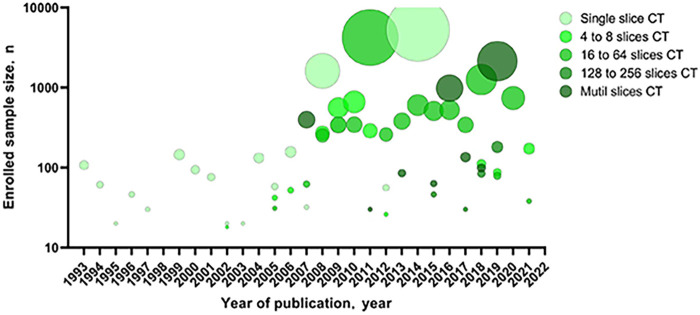
Sample size of the articles included in the systematic review by year of publication and MDCT. MDCT, multi-detector computed tomography.

The sample sizes of the recent publications were significantly larger than before, and the CT equipment was more advanced (Figure 2). Articles included a variety of breath-holding procedures, such as only inspiratory, expiratory, or both inspiratory and expiratory.

**Figure 3:**
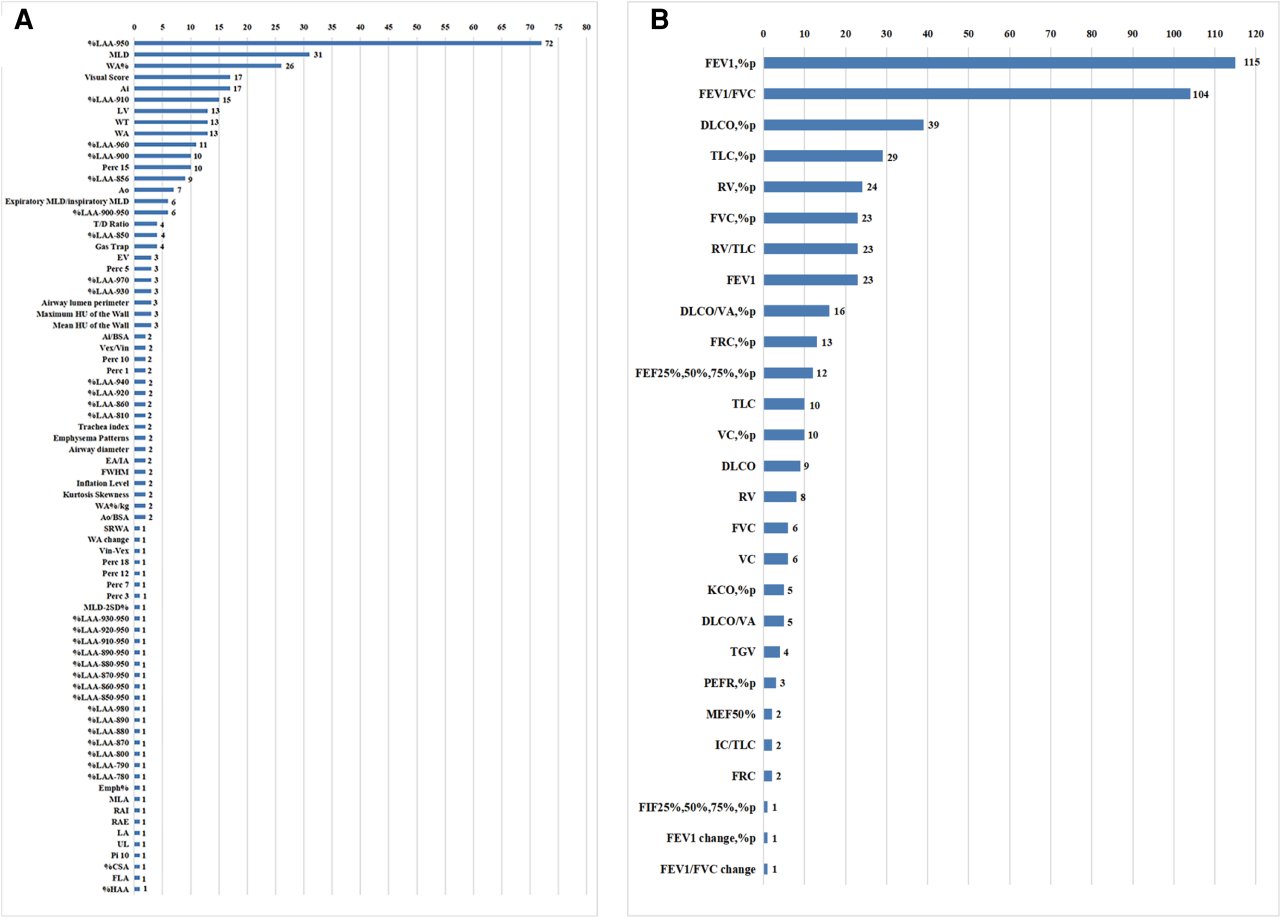
Counting of CT measurements and pulmonary function test parameters in the systematic review.

The selected articles included 79 quantitative CT parameters and 29 pulmonary function parameters (Figure 3), and the final parameters for the systemic review included %LAA-950, %LAA < 950, WA%, MLD, ATI, Perc15, WT, AI, FEV_1_%pred, and FEV_1_/FVC. The common threshold defining the lung parenchyma in emphysema was −900 to −960 HU, and the most commonly used threshold was −950 HU (19). In some study, different thresholds in the same sample had different correlations with airflow obstruction parameters in PFTs (13, 14, 15, 20-22). The results show that there is a significant correlation between quantitative CT parameters and airflow limitation parameters (Table 1).

**Table 1:** Correlation coefficients between CT measurements and airflow obstruction parameters of pulmonary function test in the systematic review.

	FEV_1_/FVC	FEV_1_%Pred
%LAA-950	−0.74 to −0.99	−0.66 to −0.77
%LAA < −950	−0.87 to −0.19	−0.91 to −0.20
%LAA-950 (GE)	–	−0.67 to −0.43
%LAA-950 (non-GE)	–	−0.62 to −0.40
WA%	−0.59 to −0.009	−0.713 to −0.044
ATI	−0.74 to −0.58	−0.70 to −0.29
WT	−0.62 to −0.05	−0.68 to −0.13
MLD	0.21–0.89	0.18–0.85
Per 15	0.12–0.61	0.09–0.62
AI	0.07–0.32	0.14–0.73

### Risk of bias in the meta-analysis

All articles included in the meta-analysis were high quality (Supplementary Tables S4, S5); QUADAS scores ranged from 12.5 to 13.5 (Supplementary Table S6). QUADAS 2 where CT density was being considered as a diagnostic tool. Funnel plots and Begg-Mazumdar/Egger tests were selected to assess publication bias and reduce bias by excluding date or language limits during our search. No publication bias was found (Supplementary Table S7 and Supplementary Figures S1, S2).

Several of the meta-analyses showed slight heterogeneity. The *I*^2^ index was > 50% for correlations between WA% and FEV_1_%pred in inspiration (*P* = 0.018, *I*^2^ index = 56.8%), ATI and FEV_1_%pred in inspiration (*P* < 0.001, *I*^2^ index = 96.8%), WT and FEV_1_/FVC in inspiration (*P* < 0.001, *I*^2^ index = 96.1%), AI and FEV_1_%pred in inspiration (*P* < 0.001, *I*^2^ index = 90.7%), and AI and FEV_1_/FVC in inspiration (*P* = 0.031, *I*^2^ index = 71.1%). Because heterogeneity was high, a sensitivity analysis was conducted to explore the sources of the heterogeneity. Finally, we found that Yamashiro's (41) study was the main source of heterogeneity mainly because this study when measuring the thickness of the bronchial wall only the third, fourth, and fifth layers of the right bronchus were selected. The result obtained is not the average value of the bilateral bronchus which may cause selection bias. After excluding Yamashiro's study, the heterogeneity became significantly lower than before. Such as, WA% and FEV_1_%pred in inspiration (*P* = 0.057, *I*^2^ index = 48.9%). Then, we found that Washko's (35) study was also the main source of heterogeneity because subjects with GOLD 3 and 4 diseases were pooled into one group (GOLD 3&4) due to limited numbers of subjects with GOLD stage 4 disease which may cause selection bias. After excluding Washko's study, the heterogeneity became significantly lower than before. AI and FEV_1_%pred in inspiration (*P* = 0.203, *I*^2^ index = 37.3%), and AI and FEV_1_/FVC in inspiration (*P* = 0.376, *I*^2^ index = 0.0%). Because there are only two studies between ATI and FEV_1_/FVC in inspiration, the heterogeneity is still high. This needs to include more study to explore the source of heterogeneity.

### Synthesis of results in the meta-analysis

**Figure 4:**
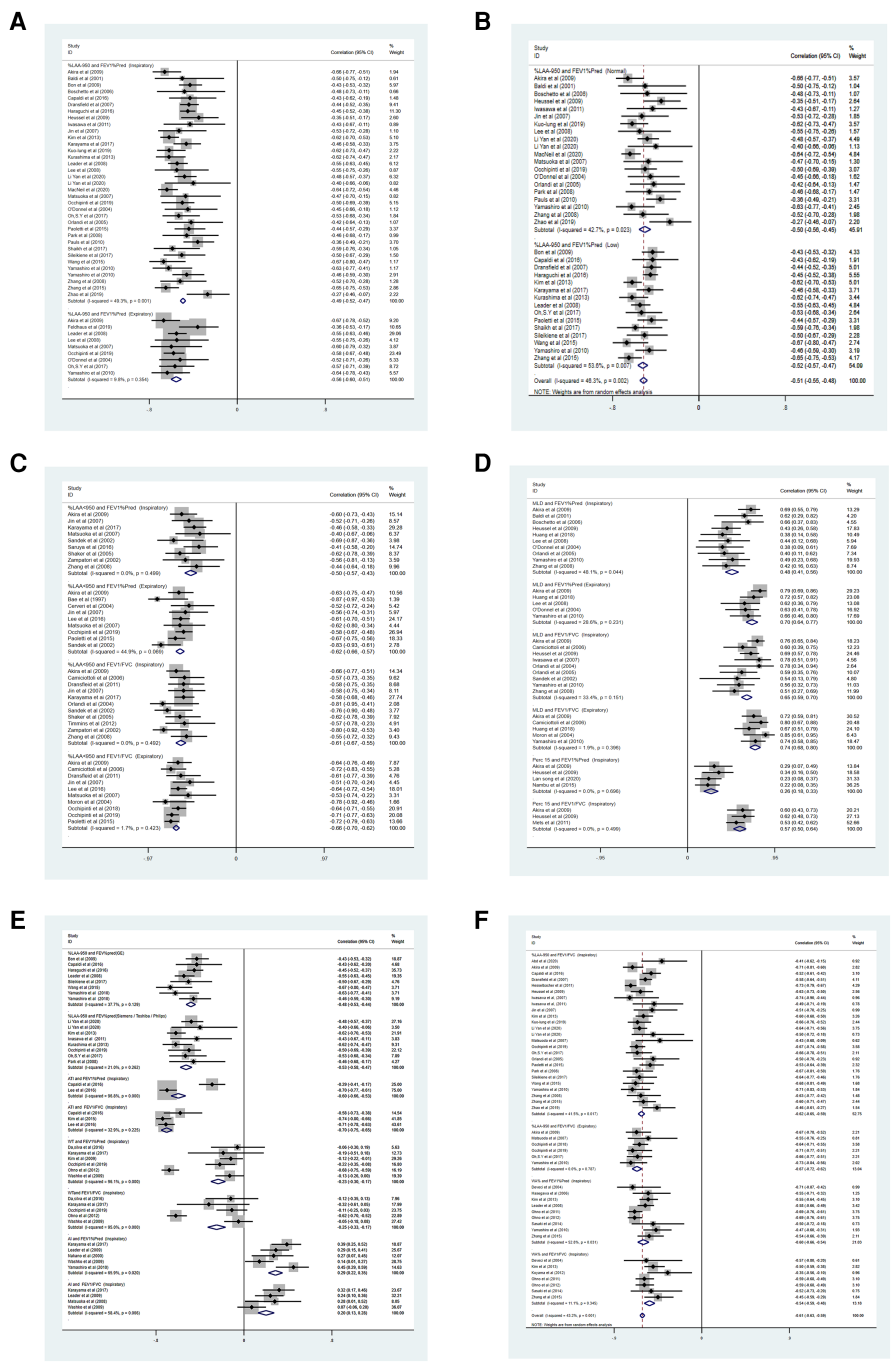
(**A–F**) Forest plots for correlations between CT measurements and airflow obstruction. CCI, confidence interval; *P*(*Z*) = *P* value of *Z* test; FEV1%pred, percentage of the predicted forced expiratory volume in the first second.

A total of 6,341 participants were included in the meta-analysis (Figure 4). The CC between %LAA-950 and FEV_1_%pred in inspiration was reported in 35 articles (10, 14, 15, 20, 21, 26, 28, 32, 37–39, 41–60, 61–64). Two National Lung Screening Test (NLST) cohort studies (35, 44) and two Korean Obstruction Lung Disease (KOLD) cohort studies (26, 58) have been performed. The pooled CC between %LAA-950 and FEV_1_%pred was −0.49 (−0.52, −0.47), −0.56 (−0.60, −0.51) in inspiration and expiration (10, 14, 15, 26, 51, 55, 56, 62, 65), respectively. The pooled CC between %LAA-950 and FEV_1_/FVC was −0.61 (−0.63, −0.58), −0.67 (−0.73, −0.61) in inspiration (10, 14, 15, 20, 21, 28, 32, 37, 39, 44, 46, 47, 49, 52, 53, 56–58, 61–64, 66–69) and expiration (10, 14, 15, 56, 62, 70), respectively. The pooled CC between %LAA < 950 and FEV_1_%pred was −0.50 (−0.57, −0.43), −0.62 (−0.66, −0.57) in inspiration (14, 15, 20, 21, 24, 29, 30, 48, 71, 72) and expiration (10, 14, 15, 21, 24, 31, 57, 73, 74), respectively. The pooled CC between %LAA < 950 and FEV_1_/FVC was −0.61 (−0.67, −0.55), −0.66 (−0.70, −0.62) in inspiration (14, 16, 20, 21, 24, 30, 48, 72, 75–77) and expiration (10, 14, 15, 21, 31, 57, 70, 75, 76, 78), respectively.

Twelve articles (25–27, 34, 36, 38, 39, 51, 62, 63, 79, 80) reported CCs between WA% and lung function tests in inspiration. Two articles (26, 38) were excluded because the airway measurements only involved the airway above the fifth generation. Therefore, a total of 10 articles were included (25, 27, 34, 36, 39, 51, 62, 63, 79, 80). In the included literature, the average lumen diameter of the peripheral airway was about 2–3 mm. The pooled CC value between WA% and FEV_1_%pred was −0.59 (95% CI: −0.63, −0.56), between WA% and FEV_1_/FVC was −0.53 (95% CI: −0.58, −0.48) in inspiration. Expiratory CT was not used for airway measurements.

The CC between MLD and FEV_1_%pred was reported in fifteen articles (14, 16, 20, 23, 24, 26, 28, 42, 43, 46, 55, 62, 75, 78, 81). The pooled CC between MLD and FEV_1_%pred was 0.48 (0.41, 0.56), 0.70 (0.64, 0.77) in inspiration (14, 20, 26, 28, 42, 43, 46, 55, 62, 81) and expiration (14, 26, 55, 62, 81), respectively. The pooled CC between MLD and FEV_1_/FVC was 0.65 (0.59, 0.70), 0.74 (0.68, 0.80) in inspiration (14, 16, 20, 23, 24, 28, 46, 62, 75) and expiration (14, 62, 75, 78, 81), respectively.

Only three studies (31–33) were analyzed according to strict criteria for inclusion, we determined that the pooled CC between ATI and FEV_1_/FVC was −0.70 (95% CI: −0.75, −0.65) in inspiration.

Five articles (14, 40, 46, 82, 83) reported CCs between Perc15 and FEV_1_%pred in inspiration; however, Perc15 and FEV_1_/FVC only had three groups of data.The pooled CC between AI and FEV_1_%pred was 0.26 (95% CI: 0.18, 0.33) in inspiration, and the pooled CC of AI and FEV_1_/FVC was 0.57 (95% CI: 0.50, 0.64) in inspiration.

Eight articles (32, 37, 38, 41, 45, 51, 61, 62) reported CCs between LAA-950 and FEV_1_%pred using GE in inspiration. Eight articles (10, 39, 50, 52, 53, 56, 58) reported CCs between LAA-950 and FEV_1_%pred using other brands of CT machines (such as Siemens, Toshiba, and Philips) in inspiration. The pooled CC between LAA-950 and FEV_1_%pred using GE was −0.50 (95% CI: −0.56, −0.45) and −0.54 (95% CI: −0.60, −0.49) between LAA-950 and FEV_1_%pred using other brands of CT machines.

### Subgroup analysis

We performed a subgroup analysis, depending on the densitometric thresholds (29). At <−950 HU (between −856 and −950 HU) thresholds, the pooled CC was −0.50 (95% CI: −0.57, −0.43). At thresholds of −950 HU, the pooled CC was −0.49 (95% CI: −0.52, −0.47). Similarly, a subgroup analysis was performed for for radiation dose was performed for the association between %LAA-950 and FEV_1_%pred in inspiration, indicating no significant difference (*P = *0.4494). In normal dose, the pooled CC was −0.50 (95% CI: −0.56, −0.45). In low dose, the pooled CC was −0.52 (95% CI: −0.57, −0.47). Subgroup analysis was performed for inspiratory and expiratory CT. Compared with inspiratory CT, expiratory CT %LAA-950 showed a stronger negative correlation with FEV_1_%pred and FEV_1_/FVC (*P* < 0.05), MLD and FEV_1_%pred showed a stronger positive correlation (*P* < 0.001), FEV_1_/FVC also shows the same trend. In addition, subgroup analyses were also performed based on the brands of CT machines. The pooled CC was −0.50 (95% CI: −0.56, −0.45) in the first group (GE) and −0.54 (95% CI: −0.60, −0.49) in the second group (Siemens, Toshiba, and Philips), showing that there was no significant difference between %LAA-950 and FEV_1_%pred according to CT machine (*P = 0.882)*.

## Discussion

In the current study, we conducted a systematical review and meta-analysis to determine the relationship between quantitative CT parameters and airflow obstruction in patients with COPD. The result of this meta-analysis suggested that there were correlations between CT measurements and airflow obstruction parameters in PFTs in patients with COPD, both in inspiratory and expiratory CT. In the included studies, the absolute CCs of CT measurements and airflow limitation were as follows: inspiratory CT, 0.44 to 0.71 and expiratory CT, 0.59 to 0.66. These results were consistent with other studies that have revealed that expiratory CT can be used as an auxiliary examination for inspiratory CT (14, 46, 84, 85). This reconfirmed our hypothesis that there was a significant correlation between the proportion of emphysema, WA%, MLD, AI, Perc15, ATI, WT, and lung function in patients with COPD. Therefore, this approach generates reproducible and sensitive measurements of COPD that are related to pulmonary ventilation and perfusion as well as the anatomical and morphological features of the airway wall and parenchyma (86).

Pulmonary function is the main objective test for determining airflow limitation. FEV_1_/FVC can detect mild airflow obstruction, which is beneficial for early detection and treatment of patients with COPD. However, lung function does not provide information on regional dysfunction. With the widespread use of quantitative CT and the continuous improvement of corresponding software, lung structure and function abnormalities can be regionally identified and measured. %LAA-950 and MLD can reflect the extent of damage to the lung parenchyma. Meanwhile, it can be used to measure the thickness of the bronchial wall to assess the degree of airflow obstruction. Thus, quantitative CT is a morphologica method for identifying morphological information regarding the degree of airway stenosis and the proportion of emphysema, which are complementary to lung function.

This systematic review included ≥ 10 different CT measurements. However, because of the insufficient number of studies, only 7 items (%LAA-950, %LAA < 950 MLD, WA%, Perc15, AI, ATI, and WT) were used in the meta-analysis. We also evaluated FEV_1_/FVC and FEV_1_%pred from PFTs because these are important factors associated with the diagnosis of COPD and the classification of airflow limitation. Furthermore, the above two parameters were relatively comprehensive and easy to extract.

We performed a subgroup analysis based on different respiratory processes (including inspiratory and expiratory processes). Our results demonstrated that CT measurements in expiratory were more strongly correlated with FEV_1_%pred and FEV_1_/FVC than inspiratory. This was consistent with other findings that have suggested that CT measurements in expiratory are more strongly correlated with airflow limitation than in inspiratory (55, 87, 88). However, the significance of expiratory CT data for the assessment of COPD still requires additional data for further study. Further, we also performed a subgroup analysis according to the brand of CT machines, and the results indicated that %LAA-950 was correlated with lung function regardless of the brand of CT machine, which was consistent with previous studies by imaging experts (19). Meanwhile, our results showed that %LAA ≤ −950 HU (between −856 and −950 HU) were more strongly correlated with FEV_1_%pred than %LAA-950 HU in expiratory. This was consistent with other findings that have suggested that the selected CT parameters (between −856 HU and −950 HU), software programs, reconstruction algorithms, and section thickness vary widely throughout the process (19). While the prolonged examination time also reminded us to perform adequate breathing training for patients before the CT examination. Also, Our study suggested low radiation doses did not change correlations between CT emphysema quantification and airflow obstruction compared to normal doses. Low-dose CT can decrease the overall radiation dose for CT quantitative emphysema evaluation without loss in diagnostic value. Quantitative CT indicators provide support for thoracic surgeons and interventional pulmonologists to select patients and optimize LVR procedures, as well as to develop new endobronchial therapies to further improve outcomes in patients with reduced lung volume (13).

Some researchers have studied the third to fifth or sixth generation airways and found that the correlation between airway wall measurements and PFTs was stronger in the smallest airways (19, 41, 43). To reduce the deviation caused by different airway generations, we unified ≥5th generation airways and included them in the meta-analysis. From our results, the correlation between WA% and FEV_1_%pred was −0.59. Based on the above results, the airway WT measurement from CT was more reliable in the smallest airway.

Although some previous meta-analyses (15, 16, 55, 85) have evaluated the relationship between quantitative CT and lung function in patients with COPD, the current meta-analyses reconfirmed these findings and had multiple advantages. First of all, this meta-analysis had a large sample size, which made our results more reliable. Second, all included studies had more quantitative CT parameters, such as WT, Prec15 and AI. Subgroup analyses were also performed according to the densitometric thresholds (%LAA-950 HU, −910 HU, and −900 HU) and the brands of CT machines. These comparisons have often been overlooked in previous studies, and we found that there are many brands of CT machines (such as GE, Siemens, Toshiba and Philips). Therefore, it is necessary to conduct subgroup analyses to determine if brands of CT machines impact the results. Third, the results of most studies were highly consistent. After sensitivity analysis and publication bias analysis, the source of heterogeneity has been found, which provides ideas for future experimental designs. Fourth, our results enriched and validated the previous conclusions.

Meanwhile, this meta-analysis inevitably had some limitations. First, the results of this study may be influenced by age, race, and the male-female ratio. Additionally, the severity of the disease varied among participants in the included studies. Second, a variety of quantitative CT parameters and PFTs parameters were extracted for systematic evaluation but only representative parameters with complete data were selected for the meta-analysis. Measurements that were not included may be valuable for the evaluation of COPD, and these measurements require further research. Above all, the interval time between lung function and quantitative CT was inconsistent, and this may have impacted the measurements of quantitative CT parameters. In addition, we used different brands of post-processing software, work stations that we did not examine in more details because their impact was considered less relevant (19). This study just conducted a subgroup analysis according to the brands of CT machines, and the results showed that quantitative CT parameters were correlated with lung function regardless of the brands of CT machines. Thus, this systematic review was based on studies of high methodological quality, and there was no publication bias; therefore, the results have a certain strength of argumentation.

## Conclusions

Results from this study provided evidence that quantitative CT parameters are significantly correlated with lung function in patients with COPD. Quantitative CT may provide a morphological approach for accurate and early diagnosis of COPD and testing new interventions and therapies for patients with COPD.

## Data Availability

The original contributions presented in the study are included in the article/**Supplementary Material**, further inquiries can be directed to the corresponding author.

## Supplementary material

Supplementary Figure S1 Funnel plot about the publication bias for Correlation coefficients between CT measurements and airflow obstruction parameters

Supplementary Figure S2 Trim-fill graph about the publication bias for Correlation coefficients between CT measurements and airflow obstruction parameters

Supplementary Table S1 Electronic supplementary table 1 Literature search strategy. Last search on July 5, 2022

Supplementary Table S2 Characteristics of studies included in the systematic review

Supplementary Table S3 Correlation coefficients between CT measurements and airflow obstruction parameters of pulmonary function test in the systematic review

Supplementary Table S4 Characteristics of studies included in the meta-analysis

Supplementary Table S5 Correlation coefficients between CT measurements and airflow obstruction parameters of pulmonary function test in the meta-analysis

Supplementary Table S6 Assessment of Study Quality, by the Quality Assessment of Diagnostic Accuracy Studies (QUADAS) tool

Supplementary Table S7 Bias analysis in the meta-analysis
