# Supplementary figures and images for: Quantitative CT parameters correlate with lung function in chronic obstructive pulmonary disease: A systematic review and meta-analysis
Source: Front Surg. 2023 Jan 4;9:1066031. doi: 10.3389/fsurg.2022.1066031 (PMC9845891; doi:10.3389/fsurg.2022.1066031)

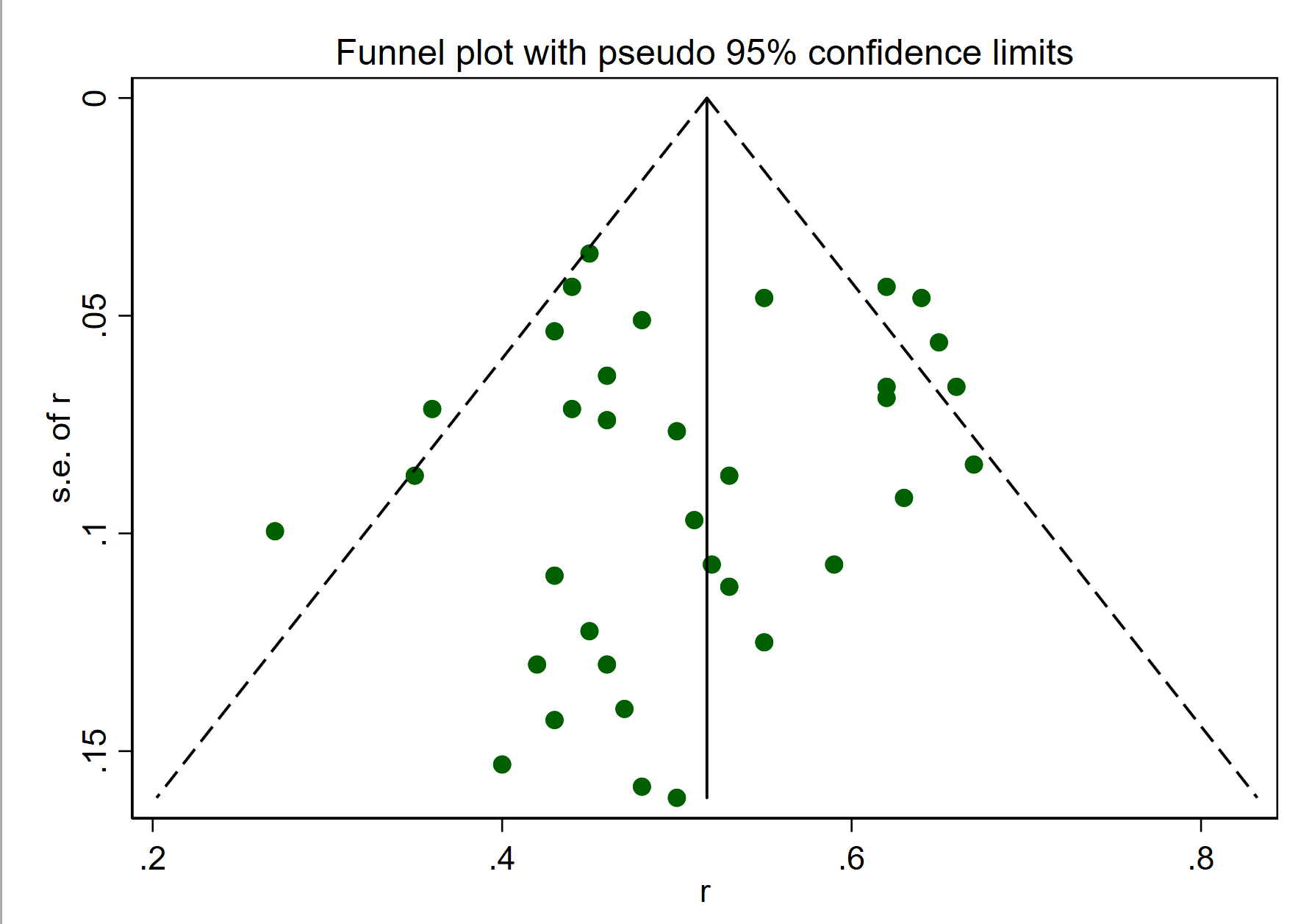

Supplement: Supplementary Figure S1 — Funnel plot about the publication bias for Correlation coefficients between CT measurements and airflow obstruction parameters [file Figure5.tif]

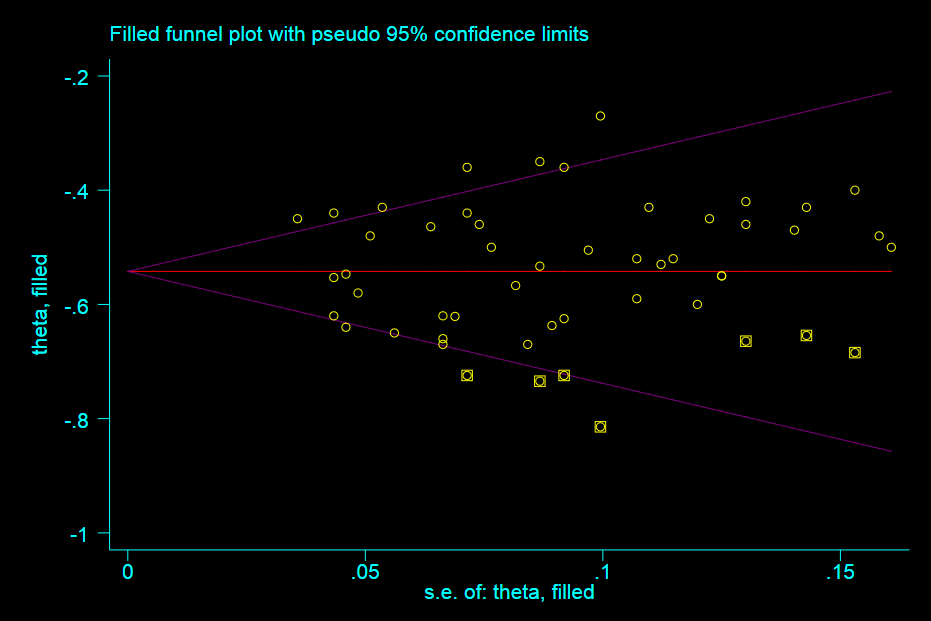

Supplement: Supplementary Figure S2 — Trim-fill graph about the publication bias for Correlation coefficients between CT measurements and airflow obstruction parameters [file Figure6.tif]
