# Supplementary material for: Quantitative CT parameters correlate with lung function in chronic obstructive pulmonary disease: A systematic review and meta-analysis
Source: Front Surg. 2023 Jan 4;9:1066031. doi: 10.3389/fsurg.2022.1066031 (PMC9845891; doi:10.3389/fsurg.2022.1066031)
Supplement: Supplementary Table S1 — Electronic supplementary table 1 Literature search strategy. Last search on July 5, 2022 [file Table1.docx]

**Electronic supplementary table 1 Literature search strategy. Last search on July 5, 2022**

**Search terms used to identify relevant citations**

| **Search terms used to identify relevant citations**  A: Computed tomography  CT  B: Lung function*  Respiratory function*  Pulmonary function*  Spirometr*  Diffusing capacity  Diffusion capacity  Airway obstruction parameter*  C: Chronic obstructive pulmonary disease*  COPD  Chronic obstructive lung disease*  Chronic airflow obstruction*  To search: A and B and C  The term ‘CT’ is restrained to the title and abstract  Publication date: From January 1976 to December 2011  **Search Query for PubMed**  #1: ("Tomography, X-Ray Computed"[MeSH] OR "computed tomography"[TIAB] OR CT[TIAB])  #2: ("Pulmonary Disease, Chronic Obstructive"[MeSH] OR "chronic obstructive pulmonary disease*"OR "chronic obstructive lung disease*" OR "Chronic airflow obstruction*"OR "Airflow Obstruction, Chronic*" OR "Chronic Obstructive Airway Disease*" OR "Airflow Obstructions, Chronic*" OR "Chronic Airflow Obstruction*"OR “COPD”)  #3: ("Pulmonary Function Test" OR Spirometr* OR "pulmonary function*" OR "Lung function*" OR "Respiratory function*" OR "Diffusing capacity" OR "Diffusion capacity" OR "Airway obstruction parameter*" OR "Respiratory Function Tests*" OR "Function Test, Respiratory*" OR "Function Tests, Respiratory*" OR "Respiratory Function Test*" OR "Test, Respiratory Function*" OR "Tests, Respiratory Function*" OR "Pulmonary Function Tests*" OR "Function Test, Pulmonary*" OR " Function Tests, Pulmonary*" OR "Tests, Pulmonary Function*" OR " Lung Function Tests*" OR " Function Test, Lung*" OR " Function Tests, Lung*" OR " Lung Function Test*" OR "Test, Lung Function*" OR " Tests, Lung Function*")  Grammar in advanced search: 1976/01:2018/12 [dp] and #1 and #2 and #3  **Search Query for Embase**  #1: ((Computed tomography*) OR (computed tomographic scan*) OR (computed tomography scan*) OR (computer tomography*) OR (computerised tomography*) OR (computerized tomography*) OR (computerized tomography scan*) OR CT ))  #2: ((Chronic Obstructive Pulmonary Disease*) OR COPD OR (Chronic Obstructive Lung Disease*) OR (Chronic obstructive airway disease*) OR (chronic airflow obstruction*) OR (chronic obstructive bronchitis*) OR (chronic obstructive bronchopulmonary disease*) OR (chronic obstructive lung disorder*) OR (chronic obstructive pulmonary disorder*) OR (chronic obstructive respiratory disease*) OR (obstructive respiratory tract disease*) OR (pulmonary disorder, chronic obstructive*) OR (Chronic airflow obstruction*))  #3: ('lung function test'/exp OR (Spirometr*) OR ('Lung function') OR ('Pulmonary function') OR ('Respiratory function') OR ('Lung functions') OR ('Pulmonary functions') OR ('Respiratory functions'') OR ('respiratory test'') OR ('ventilation test ') OR ('Diffusing capacity') OR ('Diffusion capacity') OR ('Airway obstruction'))  Grammar in advanced search: #1 AND #2 AND #3 AND [1-1-1976]/sd NOT (  **Search Query for Web of Knowledge**  #1 topic: ((Computed tomography) OR CT)  #2 topic: ((Chronic Obstructive Pulmonary Disease*) OR COPD OR (Chronic Obstructive Lung Disease*) OR (Chronic obstructive airway disease*) OR (Chronic airflow obstruction*))  #3 topic: (Spirometr* OR (Lung function*) OR (Pulmonary function*) OR (Respiratory function*) OR (Diffusing capacity) OR (Diffusion capacity) OR (Airway obstruction parameter*))  Grammar: #1 topic and #2 topic and #3 topic  **Search Query for Cochrane**  #1: ((Multidetector-Row Computed Tomography) OR (Multidetector Row Computed Tomography) OR (Computed Tomography, Multisection) OR (Multislice Computed Tomography) OR (Computed Tomography, Multidetector))  #2: ((Pulmonary Disease, Chronic Obstructive) OR (Chronic Airflow Obstruction) OR (Airflow Obstructions,Chronic) OR (Chronic Airflow Obstructions) OR (Airflow Obstruction, Chronic) OR (Chronic Obstructive Pulmonary Disease) OR (Chronic Obstructive Lung Disease) OR (COAD) OR (Chronic Obstructive Airway Disease) OR (COPD) OR (Chronic Obstructive Pulmonary Disease))  #3:((Respiratory Function Tests) OR (Test, Respiratory Function) OR (Respiratory Function Test) OR (Function Test, Respiratory) OR (Function Tests, Respiratory) OR (Tests, Respiratory Function) (Function Tests, Pulmonary) OR (Lung Function Test) OR (Test, Lung Function) OR (Tests, Pulmonary Function) OR (Pulmonary Function Tests) (Lung Function Tests) OR (Function Test, Lung) OR (Test, Pulmonary Function) OR (Tests, Lung Function) OR (Function Test, Pulmonary) OR (Pulmonary Function Test) OR (Function Tests, Lung) OR (Test, Pulmonary Function) OR (Tests, Lung Function) OR (Function Test, Pulmonary))  Grammar in advanced search: #1 and #2 and #3 |
| --- |
