# Supplementary material for: Quantitative CT parameters correlate with lung function in chronic obstructive pulmonary disease: A systematic review and meta-analysis
Source: Front Surg. 2023 Jan 4;9:1066031. doi: 10.3389/fsurg.2022.1066031 (PMC9845891; doi:10.3389/fsurg.2022.1066031)
Supplement: Supplementary Table S2 — Characteristics of studies included in the systematic review [file Table2.docx]

**Electronic supplementary table 2 Characteristics of studies included in the systematic review**

| **Study, Year** | **Patients, *n*** | **Men, %** | **Age,**  **Year ± SD or**  **year (range)** | **COPD Severity,**  **GOLD stage or**  **FEV_1_ %pred ± SD or FEV_1_ %pred (range)** | **CT type, slice** | **Inspiratory or expiratory CT examination** | **Volumetric CT examination** | **Radiation Dose** | **Cohort Name** |
| --- | --- | --- | --- | --- | --- | --- | --- | --- | --- |
| Abd et al, 2020[S1] | 50 | 100 | 62.82±8.65 | Stage0-IV | 16 | Inspiratory | Volume | Normal |  |
| Achenbach et al, 2008 [S2] | 16 | 75 | 65(50-75) | Stage II-III | 4 | Inspiratory | Volume | Normal |  |
| Akira et al, 2009* [14] | 76 | 88 | 67(37-85) | Stage 0-IV | 16 | Both | Volume | Normal |  |
| Bae et al, 1997* [68] | 10 | NA | 57(41-77) | NA | 1 | Both | Non-volume | Normal |  |
| Bafadhel et al, 2011 [S3] | 75 | 77 | 67(43-88) | 47±2%^†^ | 16 | Inspiratory | Non-volume | Normal | BCOPDE |
| Bai et al, 2015 [S4] | 63 | 59 | 53±8.9 | Stage II-IV | Multi | Both | Volume | Low |  |
| Baldi et al, 2001* [73] | 24 | 75 | 61±11 | 35%(17-72%) | 1 | Inspiratory | Non-volume | Normal |  |
| Beinert et al, 1995 [S5] | 11 | 82 | 48(34-56) | 41±0.04%^†^ | 1 | Both | Non-volume | Normal |  |
| Bon et al, 2009* [21] | 234 | 50 | 61(50-78) | Stage 0-IV | 4&8 | Inspiratory | Volume | Low | PLuSS |
| Boschetto et al, 2006* [74] | 26 | 85 | 71±3(*n*=11)^†^  70±2(*n*=15)^†^ | 31±2.6%(*n*=11)^†^  47±3.8%(*n*=15)^†^ | 1 | Inspiratory | Non-volume | Normal |  |
| Camiciottoli et al, 2006* [84] | 51 | 90 | 64(43-78) | 52%(15-106%) | 1 | Both | Non-volume | Normal |  |
| Camiciottoli et al, 2012 [S6] | 72 | 74 | 66±8 | Stage II-IV | 16 | Both | Non-volume | Low |  |
| Capaldi et al, 2016* [36] | 58 | 69 | 73±9 | Stage I-IV | 64 | Both | Volume | Low |  |
| Cavigli et al, 2009 [S7] | 30 | 77 | 68(52-81) | Stage I-IV | 16 | Inspiratory | Volume | Normal |  |
| Cerveri et al, 2004 [S8] | 39 | 90 | 64(48-80) | 47%(21-72%) | 1 | Expiratory | Non-volume | Normal |  |
| Charbonnier et al, 2019[S9] | 2046 | 50.8 | 60.5±8.9 | Stage0-IV | NA | Inspiratory | Volume | Normal |  |
| Crausman et al, 1995 [S10] | 9 | 22 | 67±2^†^ | 40%(20-89%) | 1 | Inspiratory | Non-volume | Normal |  |
| D’Anna et al, 2011 [S11] | 59 | 68 | 68±7 | 52±18 | 16 | Inspiratory | Non-volume | Normal |  |
| Da,silva et al, 2016 [S12] | 65 | 66 | 64.15±8.45 | Stage III-IV | 64 | Both | Volume | Normal |  |
| Daghfous et al, 1993 [S13] | 51 | 90 | 55(25-80) | 40±21%(*n*=31)  39±17%(*n*=20) | 1 | Inspiratory | Non-volume | NA |  |
| Demir et al, 2005 [S14] | 16 | 100 | 65±6 | 41±15% | 1 | Inspiratory | Non-volume | NA |  |
| Deveci et al, 2004 [65] | 22 | 100 | 57±5(*n*=17)  61±7(*n*=5) | Stage II-III | 1 | Inspiratory | Non-volume | Normal |  |
| Dransfield et al, 2007* [22] | 396 | 62 | 63±5(*n*=246)  61±5(*n*=150) | Stage 0-IV | Multi | Inspiratory | Volume | Low | NLST |
| Dransfield et al, 2011 [S15] | 46 | 56 | 67.7±7.9 | Stage 0-III | 16 | Both | Volume | Low |  |
| Falaschi et al, 1995 [63] | 46 | 83 | 63(46-78) | NA | 1 | Both | Non-volume | Normal |  |
| Feldhaus et al, 2019[S16] | 88 | 60 | 66±6.6(45-79) | StageIII-IV | NA | Both | Volume | Normal |  |
| Fuseya et al, 2018 [S17] | 40 | NA | 70.5(65.0-76.8) | NA | 64 | Inspiratory | Non-volume | Low |  |
| Gawlitza et al, 2018 [S18] | 46 | 57 | 66±10 | NA | 4 | Both | Volume | Both |  |
| Gawlitza et al, 2018* [S37] | 65 | NA | NA | Stage I-IV | 4 | Both | Volume | Low |  |
| Gelb et al, 1993 [S19] | 56 | 48 | 68(53-76) | 46%(18-79%) | 1 | Inspiratory | Non-volume | Normal |  |
| Grydeland et al, 2010 [S20] | 463 | 65 | 65±9(*n*=299)  63±9(*n*=164) | Stage II-IV | 8 | Inspiratory | Non-volume | Normal | GenKOLS |
| Grydeland et al, 2011 [S21] | 288 | 70 | 64±10(*n*=202)  60±8(*n*=86) | Stage II-IV | 8 | Inspiratory | Non-volume | Normal | GenKOLS |
| Haraguchi et al, 2016* [47] | 443 | 92 | 72.6±8.2 | Stage I-IV | Multi | Inspiratory | Volume | Low |  |
| Hasegawa et al, 2006*[57] | 52 | 96 | 72(41-84) | Stage I-IV | 4 | Inspiratory | Volume | Normal | Hokkaido |
| Hesselbacher et al, 2011* [32] | 224 | 65 | >40 | Stage I-IV | 64 | Inspiratory | Volume | Normal | LESCOPD |
| Heussel et al, 2009* [75] | 102 | NA | 64(20-87) | Stage III-IV | 4 | Inspiratory | Volume | Normal |  |
| Hoshino et al, 2014 [39] | 54 | NA | >40 | Stage I-IV | 64 | Inspiratory | Non-volume | Normal |  |
| HUANG Xiao-qi et al, 2018 [85] | 60 | 78 | 47-78 | StageI-IV | 128 | Both | Volume | Normal |  |
| Hyun Jung Koo et al, 2019[12] | 370 | 97 | 65-75 | StageI-IV | 16&64 | Both | Volume | Normal |  |
| Iwasawa et al, 2007* [64] | 19 | 100 | 71±7 | Stage II-IV | 16 | Inspiratory | Volume | Normal |  |
| Iwasawa et al, 2011* [34] | 35 | 100 | 70±6 | Stage I-IV | 16 | Inspiratory | Volume | Normal |  |
| Jin et al, 2007* [35] | 43 | 51 | 65(45-85) | NA | 16 | Both | Volume | Normal |  |
| Jogi et al, 2011 [S22] | 30 | 65 | 65(53-76) | 51%(25-81%) | Multi | Inspiratory | Volume | NA |  |
| Ju et al,2014* [40] | 350 | 57 | 64 | Stage 0-IV | 64 | Inspiratory | Non-volume | Low |  |
| Karayama et al, 2017* [51] | 147 | 93 | 73 | Stage I-IV | 64 | Inspiratory | Volume | Low |  |
| Kaya et al, 2017 [S23] | 30 | 96 | 41-72 | Stage I-III | 128 | Both | Volume | Normal |  |
| Kim et al, 2009 [67] | 338 | 64 | 68±6 | <45% | 1 | Inspiratory | Non-volume | Normal | NETT |
| Kim et al, 2011 [S24] | 842 | 58 | 53.8±6.9(n=507) 59.2±7.8(n=335) | Stage II-IV | 16&64 | Both | Volume | Low |  |
| Kim et al, 2013* [42] | 200 | 64 | 64.6±8.4 | Stage I-IV | 16&64 | Inspiratory | Volume | Low |  |
| Kim et al, 2014 [62] | 78 | 57 | 61.8±8.5 | Stage II-IV | 16 | Inspiratory | Non-volume | Normal |  |
| Kim et al, 2015* [43] | 167 | 89 | 64.8±8.2(n=138) 51.7±9.4(n=29) | Stage 0-IV | 16 | Both | Non-volume | Low |  |
| Kirby et al, 2017 [S25] | 135 | 30 | 67.5 | Stage I-II | Multi | Both | Volume | Low |  |
| Kosciuch et al, 2009 [S26] | 12 | 58 | 57±9 | 72±19% | 16 | Inspiratory | Non-volume | Normal |  |
| Koyama et al, 2012* [87] | 56 | 71 | 65.5(32-85) | Stage I-IV | 1 | Inspiratory | Volume | Low |  |
| Kundu et al, 2013 [44] | 180 | 52 | 65.6±9.7 | Stage 0-IV | 16 | Both | Non-volume | Normal |  |
| Kuo-Lung Lor et al, 2019*[76] | 87 | 97 | 67.3 | StageI-IV | 4 | Inspiratory | Volume | Normal |  |
| Kurashima et al, 2013* [45] | 85 | 100 | 70.8±6.7 | Stage I-IV | Multi | Inspiratory | Volume | Low |  |
| Lamers et al, 1994 [S27] | 40 | 83 | 60±8(*n*=20)  70±7(*n*=20) | 46±12%(*n*=20)  51±14%(*n*=20) | 1 | Both | Non-volume | Normal |  |
| Lan Song et al, 2020*[72] | 172 | 57.6 | 65.9±6.7 | StageIII-IV | NA | Both | Volume | Low |  |
| Leader et al, 2008*[77] | 240 | NA | NA | Stage 0-IV | 4(*n*=112)  8(*n*=128) | Inspiratory | Volume | Low | PLuSS |
| Leader et al, 2009 [S28] | 200 | NA | NA | Stage 0-IV | 64 | Inspiratory | Volume | Normal | PLuSS |
| Lee et al, 2008* [25] | 34 | 97 | 65(50-78) | 45%(17-82%) | 16 | Both | Volume | Normal | KOLD |
| Lee et al, 2011 [S29] | 197 | 96 | 67±7 (*n*=126)  65±8(*n*=71) | 44±15%(*n*=126)  55±15%(*n*=71) | 16 | Both | Volume | Normal | KOLD |
| Lee et al, 2011 [S30] | 115 | NA | 65 | Stage I-IV | 16 | Both | Volume | Normal | KOLD |
| Lee et al, 2016* [46] | 174 | 91 | 62.6±9.8 | Stage 0-IV | 16 | Both | Volume | Low | KOLD |
| Li et al, 2009 [S31] | 24 | 75 | 56(32-70) | 56±16% | 64 | Both | Non-volume | Normal |  |
| LI et al, 2019[S32] | 79 | 88 | 64.57±7.80 | Stage0-IV | 64 | Both | Volume | Normal |  |
| Li Yan et al, 2020*[78] | 248 | NA | 47-79 | StageI-IV | 64 | Inspiratory | Volume | Normal |  |
| Li Yan et al, 2020*[79] | 32 | 84 | 65.8±10.55 | StageII-III | 64 | Inspiratory | Volume | Normal |  |
| MacNeil, et al, 2020*[80] | 175 | 61.7 | 69±9 | Stage0-IV | 64 | Inspiratory | Volume | Normal |  |
| Madani et al, 2010 [S33] | 16 | 63 | 62(48-83) | Stage I-IV | 4 | Both | Non-volume | Normal |  |
| Marquez-Martin et al, 2011 [S34] | 64 | NA | 64±7 | Stage I-IV | NA | Inspiratory | Non-volume | Normal |  |
| Matin et al, 2017 [S35] | 22 | 68 | 66.6±7.3 | Stage II-IV | 16 | Inspiratory | Volume | Normal |  |
| Matsuoda et al, 2007 [S36] | 32 | 88 | 73(57-89) | Stage 0-IV | 1 | Both | Non-volume | Normal |  |
| Matsuoda et al, 2008* [58] | 50 | 80 | 70(57-89) | Stage I-IV | 64 | Both | Volume | Normal |  |
| Matsuoda et al, 2008 [S37] | 36 | 86 | 71(57-89) | Stage I-IV | 64 | Both | Volume | Normal |  |
| Mets et al, 2012*[88] | 198 | NA | about 60 | Stage I-IV | 16 | Both | Volume | Low | NELSON |
| Mishima et al, 1999 [S38] | 72 | NA | NA | NA | 1 | Inspiratory | Non-volume | Both |  |
| Mochizuki et al, 2019[S39] | 133 | 74.4 | 70(61-75) | StageI-III | NA | Inspiratory | Volume | Normal |  |
| Mohamed Hoesein et al, 2011 [S40] | 2085 | 100 | 60±5 | Stage 0-III | 16 | Inspiratory | Volume | Low | NELSON |
| Moron et al, 2004* [86] | 16 | 63 | 62±9 | 40±18% | 1 | Inspiratory | Non-volume | NA |  |
| Moroni et al, 2001 [S41] | 20 | 95 | 63(42-73) | NA | 1 | Both | Non-volume | Normal |  |
| Myung H C et al,2018[S42] | 646 | 60 | 54.9(20-90) | NA | 64 | Inspiratory | Volume | Normal |  |
| Nakano et al, 1999 [S43] | 73 | NA | 69±6 | 46±20% | 1 | Inspiratory | Non-volume | Normal |  |
| Nakano et al, 2000 [S44] | 94 | NA | NA | 48%(8-124%) | 1 | Inspiratory | Non-volume | Normal |  |
| Nambu et al, 2015* [89] | 199 | NA | 64.1±8.4 | Stage II-IV | 64 | Both | Volume | Low |  |
| Nishio et al, 2016* [S45] | 30 | 83 | 70.1±12.1 | Stage I-IV | 16 | Inspiratory | Volume | Low |  |
| Nishio M et al, 2018[S46] | 87 | 77 | 67.4±11.0 | StageI-III | 320 | Inspiratory | Volume | Normal |  |
| O’Doganay et al, 2019*[38] | 12 | 33 | 65.6±5.0(M)  61.5±10.8(F) | StageI-IV | Multi | Inspiratory | Volume | Normal |  |
| O'Donnel et al, 2004* [59] | 44 | NA | 50±7(*n*=17)  57±7(*n*=10)  55±7(*n*=17) | Stage 0-IV | 1 | Both | Non-volume | Normal |  |
| Occhipinti et al, 2018* [48] | 202 | 78 | 70.3±8.1 | Stage I-IV | 64 | Both | Volume | Both |  |
| Occhipinti et al, 2019*[10] | 194 | 79 | 70±8 | StageII-III | 64&  128 | Both | Volume | Normal |  |
|  |  |  |  |  |  |  |  |  |  |
| Oelsner et al, 2016 [S47] | 538 | 54 | 63.1(45-84) | Stage I-III | Multi | Inspiratory | Volume | Both |  |
| Oh,S.Y et al, 2017* [49] | 72 | 91 | 63.6(45-79) | Stage II-IV | 16 | Both | Volume | Low |  |
| Ohara et al, 2006 [S48] | 30 | 100 | 69±8 | 41±16% | 1 | Inspiratory | Non-volume | Normal |  |
| Ohno et al, 2011* [33] | 186 | 65 | (23-87) | Stage 0-IV | 16&  64 | Inspiratory | Volume | Normal |  |
| Ohno et al, 2012* [50] | 187 | 6.5 | 23-87 | Stage 0-IV | 16&  64 | Inspiratory | Volume | Low |  |
| Orlandi et al, 2004* [16] | 11 | 82 | 68(60-75) | NA | 1 | Inspiratory | Non-volume | Both |  |
| Orlandi et al, 2005* [70] | 42 | 88 | 63(42-73) | 49%(15-83%) | 1 | Inspiratory | Non-volume | Normal |  |
| Paoletti et al, 2015* [81] | 132 | 78 | 66±8 | Stage II-IV | 64 | Both | Volume | Low |  |
| Park et al, 2008* [26] | 39 | 92 | 66(51-79) | 44±15% | 16 | Inspiratory | Volume | Normal | KOLD |
| Patel et al, 2008 [S49] | 519 | 51 | 58±5 | <60% | 1 | Inspiratory | Non-volume | Normal | ICGN |
| Pauls et al, 2010* [27] | 145 | NA | NA | Stage I-IV | 16 | Inspiratory | Volume | Normal |  |
| Pescarolo et al, 2008 [S50] | 43 | 58 | 62(44-81) | Stage 0-IV | 16&  64 | Inspiratory | Volume | Normal |  |
| Petersen et al, 2010 [S51] | 152 | NA | NA | ≥Stage II | 16 | Inspiratory | Volume | Low | DLCST |
| Sandek et al, 2002* [69] | 20 | 40 | 60±8 | 38±16% | 1 | Both | Non-volume | Normal |  |
| Saruya et al, 2016* [41] | 74 | 85 | 73(47-90) | Stage I-IV | 64 | Inspiratory | Volume | Low |  |
| Sasaki et al, 2014* [52] | 32 | 84 | 70.0±6.8 | Stage 0-IV | 32 | Inspiratory | Non-volume | Normal |  |
| Scichilone et al, 2008 [S52] | 15 | NA | 69(53-90) | Stage I-IV | 40 | Inspiratory | Volume | Normal |  |
| Shah et al, 2020[S53] | 48 | 58 | 52.27±13.30  (40-80) | StageI-IV | 64 | Inspiratory | Volume | Normal |  |
| Shaikh et al, 2017* [82] | 41 | 83 | 62.53(42-82) | Stage III-IV | 64 | Inspiratory | Volume | Low |  |
| Shaker et al, 2005* [71] | 42 | 38 | 63±8 | 48±13% | 4 | Inspiratory | Volume | Low |  |
| Shuyi Qin et al, 2021[S54] | 38 | 66 | 55-78 | Stage0-IV | 64 | Inspiratory | Volume | Normal |  |
| Sileikiene et al, 2017* [53] | 59 | 88 | 68.36±11.13 | Stage I-IV | 64 | Inspiratory | Volume | Low |  |
| Sorensen et al, 2010 [S55] | 20 | NA | 64(49-80) | 57%(37-76%) | 4 | Inspiratory | Volume | Normal |  |
| Spiropoulos et al, 2003 [S56] | 20 | 90 | 59±9 | 57±26% | 1 | Both | Non-volume | NA |  |
| Suzuki et al, 2020[S57] | 46 | 97.8 | 67.2±7.8 | Stage0-I | 64 | Inspiratory | Volume | Normal |  |
| Timmins et al, 2012* [54] | 26 | 61 | 69.6±8.0 | Stage I-III | 4 | Both | Volume | Low |  |
| Torres et al, 2011 [S58] | 115 | 84 | 63±10 | 75±15% | 64 | Inspiratory | Volume | Low |  |
| Tsushima et al, 2010 [S59] | 48 | 83 | 61±9 | Stage I-III | 4 | Inspiratory | Non-volume | Low | Azumi |
| Van Der Lee et al, 2006 [S60] | 50 | 58 | 60(29-83) | 46±24% | 1 | Inspiratory | Non-volume | Normal |  |
| Wang et al, 2015* [55] | 46 | 80 | 67.0±10.84 | Stage I-IV | 64&  128 | Inspiratory | Volume | Low |  |
| Washko et al, 2008 [13] | 1094 | 61 | 67±6 | <45% | 1 | Inspiratory | Non-volume | Normal | NETT |
| Washko et al, 2009* [28] | 224 | 42 | 62±5 | Stage I-IV | 4 | Inspiratory | Volume | Low | NLST |
| Washko et al, 2014 [61] | 5271 | 56 | 62.7±9(n=92) 60.4±9.2（n=5179) | Stage 0-IV | 1 | Both | Volume | Low |  |
| Watanuki et al, 1994 [S61] | 21 | NA | 65(38-77) | <70% | 1 | Inspiratory | Non-volume | NA |  |
| Xia et al, 2020[S62] | 141 | 75 | ＜60（n=68）  ＞60（n=73） | Stage0-II | 64 | Both | Volume | Normal |  |
| Xie et al, 2016 [S63] | 125 | 74 | 65.8±9.3 | Stage II-IV | 64 | Inspiratory | Volume | Low |  |
| Yahaba et al, 2014* [56] | 91 | NA | 68.9±7.9 | Stage I-IV | 64 | Both | Non-volume | Normal |  |
| Yamashiro et al, 2010* [29] | 46 | 57 | 68(46-81) | Stage 0-IV | 16 | Both | Volume | Normal | LTRC |
| Yamashiro et al, 2010* [31] | 114 | 57 | 62(56-74) | Stage I-IV | 4 | Inspiratory | Volume | Low | NLST |
| Yamashiro et al, 2011 [S64] | 46 | 57 | 68(46-81) | Stage 0-IV | 16 | Both | Volume | Normal | LTRC |
| Zampatori et al, 1997 [S65] | 20 | 80 | 69(61-86) | 33%(21-57%) | 1 | Both | Non-volume | Normal |  |
| Zampatori et al, 2001 [S66] | 15 | 80 | 63 | 32%(22-63%) | 1 | Inspiratory | Non-volume | Normal |  |
| Zampatori et al, 2001 [S67] | 17 | 77 | 66(47-78) | 47±25% | 1 | Inspiratory | Non-volume | Normal |  |
| Zampatori et al, 2002 [S9] | 18 | 67 | 61(27-81) | <71% | 4 | Inspiratory | Non-volume | Normal |  |
| Zaporozhan et al, 2005 [S68] | 31 | 71 | 60(41-76) | Stage II-IV | 16 | Both | Volume | Normal |  |
| Zhang D et al, 2018[S69] | 24 | 62.5 | 62±8(48-77) | StageI-II | 256 | Both | Volume | Normal |  |
| Zhang et al, 2008* [30] | 50 | 66 | 67±10 | Stage 0-IV | 16 | Inspiratory | Volume | Normal |  |
| Zhang et al, 2015* [23] | 112 | 60 | 66.97±10.46 | Stage 0-IV | 64 | Inspiratory | Volume | Low |  |
| Zhao et al, 2019*[83] | 87 | 71.2 | 46.35±6.56 | StageI-III | 128 | Both | Volume | Normal |  |

SD = Standard deviation; SE = Standard error; GOLD = The Global Initiative for Chronic Obstructive Lung Disease; FEV_1_ %pred = Predicted forced expiratory volume in the first second; PFT = Pulmonary function test; NA = Not available; VC = Vital capacity; BCOPDE = Biomarkers in COPD exacerbation; PLuSS = the Pittsburgh Lung Screening Study; NLST = the National Lung Screening Trial; GenKOLS = the Genetic COPD Study; LES-COPD = Longitudinal Exacerbation Study of COPD; NETT = the National Emphysema Treatment Trial; KOLD = the Korean Obstructive Lung Disease; NELSON = the Dutch-Belgian Lung Cancer Screening Trial; ICGN = the International COPD Genetics Network; DLCST = the Danish Lung Cancer Screening Trial; LTRC = the National Heart, Lung and Blood Institute Lung Tissue Research Consortium.

* Included in the meta-analysis.

† Expressed as mean ± standard error.
