# Supplementary material for: Quantitative CT parameters correlate with lung function in chronic obstructive pulmonary disease: A systematic review and meta-analysis
Source: Front Surg. 2023 Jan 4;9:1066031. doi: 10.3389/fsurg.2022.1066031 (PMC9845891; doi:10.3389/fsurg.2022.1066031)
Supplement: Supplementary Table S3 — Correlation coefficients between CT measurements and airflow obstruction parameters of pulmonary function test in the systematic review [file Table3.docx]

**Electronic supplementary table 3 Correlation coefficients between CT measure -ments and airflow obstruction parameters of pulmonary function test in the systematic review**

| **Study, Year** | **CT Measurements** | **Correlation coefficients between CT measurements and airflow obstruction, *r*** | |
| --- | --- | --- | --- |
|  |  | **Inspiratory scan** | **Expiratory scan** |
| Abd et al,2020 | LAA% | LAA% and FEV_1_:-0.313  LAA% and FVC:-0.272  LAA% and FEV_1_/FVC:-0.413 | NA |
| Achenbach et al, 2008 [42] | WA%, WA | WA% and FEV_1_ %pred : -0.537  WA and FEV_1_ %pred : -0.423 | NA |
| Akira et al, 2009 [13]* | %LAA-950, -910, MLD, Perc15 Visual score, etc. | %LAA-950 and FEV_1_ %pred: -0.659  %LAA-950 and FEV_1_/FVC: -0.712  %LAA-910 and FEV_1_ %pred: -0.601  %LAA-910 and FEV_1_/FVC: -0.661  MLD and FEV_1_ %pred: 0.694  MLD and FEV_1_/FVC: 0.764  Perc15 and FEV_1_ %pred: 0.292  Perc15 and FEV_1_/FVC: 0.600 | %LAA-950 and FEV_1_ %pred: -0.668  %LAA-950 and FEV_1_/FVC: -0.666  %LAA-910 and FEV_1_ %pred: -0.632  %LAA-910 and FEV_1_/FVC: -0.642  MLD and FEV_1_ %pred: 0.792  MLD and FEV_1_/FVC: 0.721  Perc15 and FEV_1_ %pred: 0.352  Perc15 and FEV_1_/FVC: 0.544 |
| Bae et al, 1997 [S1] | %LAA-900 | %LAA-900 and FEV_1_ %pred: -0.52  %LAA-900 and FEV_1_/FVC: -0.33 | %LAA-900 and FEV_1_ %pred: -0.87  %LAA-900 and FEV_1_/FVC: -0.45 |
| Bafadhel et al, 2011 [S2] | Emphysema pattern, WT | No CT quantitative measurements | NA |
| Bai et al, 2015 | %LAA-950 | %LAA-950 and FEV_1_ %pred: -0.205  %LAA-950 and FEV_1_/FVC: -0.106 | NA |
| Baldi et al, 2001 [S3] | %LAA-950, MLD | %LAA-950 and FEV_1_ %pred: -0.50  MLD and FEV_1_ %pred: 0.62 | NA |
| Beinert et al, 1995 [S4] | MLD | No reports on the correlation between MLD and FEV_1_ %pred or FEV_1_/FVC | NA |
| Bon et al, 2009 [18]* | %LAA-950, WA% | %LAA-950 and FEV_1_ %pred: -0.43  WA% and FEV_1_ %pred : -0.39 | NA |
| Boschetto et al, 2006 [43] | %LAA-950, MLD | %LAA-950 and FEV_1_ %pred: -0.480  MLD and FEV_1_ %pred: 0.659 | NA |
| Camiciottoli et al, 2006 [39] | %LAA-950, %LAA-910, MLD | %LAA-910 and FEV_1_/FVC: -0.57  MLD and FEV_1_/FVC: 0.60 | %LAA-910 and FEV_1_/FVC: -0.72  MLD and FEV_1_/FVC: 0.80 |
| Camiciottoli et al, 2012 | MLA,RAI,MLA,RAE | MLA and FEV_1_%Pred:0.33,RAI and FEV_1_%Pred:0.57 | MLA and FEV_1_%Pred:0.71,RAI and FEV_1_%Pred:0.68 |
| Capaldi et al, 2016* | Gas Trap,%LAA-950 | Gas Trap and FEV_1_%Pred:-0.29,Gas Trap and FEV_1_/FVC:-0.58,%LAA-950 and FEV_1_%Pred:-0.43,%LAA-950 and FEV_1_/FVC:-0.52 | No reports on the correlation to FEV1%pred or FEV1/FVC |
| Cavigli et al, 2009 [S5] | %LAA-950, MLD, Perc15, etc. | No reports on the correlation to FEV_1_ %pred or FEV_1_/FVC | NA |
| Cerveri et al, 2004 [S6] | %LAA-900 | NA | %LAA-900 and FEV_1_ %pred: -0.52 |
| Crausman et al, 1995 [S7] | %LAA-900 | %LAA-900 and FEV_1_ %pred: 0.34^†^  %LAA-900 and FEV_1_/FVC: 0.46^†^ | NA |
| D’Anna et al, 2011 [S9] | Visual Score | No CT quantitative measurements | NA |
| Da,silva et al,2016 | WT,%LAA,Gas Trap | WT and FEV_1_%Pred:-0.06,WT and FEV_1_/FVC:-0.12,Gas Trap and FEV_1_%Pred:-0.06,Gas Trap and FEV_1_/FVC:-0.02,%LAA and FEV_1_%Pred:-0.13,%LAA and FEV_1_/FVC:-0.29 | NA |
| Daghfous et al, 1993 [S8] | Visual Score | No CT quantitative measurements | NA |
| Demir et al, 2005 [S10] | Visual Score | No CT quantitative measurements | NA |
| Deveci et al, 2004 [34] | WA%, T/D ratio | WA% and FEV_1_ %pred: -0.713  WA% and FEV_1_/FVC: -0.573  T/D ratio and FEV_1_ %pred: -0.735  T/D ratio and FEV_1_/FVC: -0.579 | NA |
| Dransfield et al, 2007 [19]* | %LAA-950 | %LAA-950 and FEV_1_ %pred: -0.44 (total)  %LAA-950 and FEV_1_/FVC: -0.58 (total)  %LAA-950 and FEV_1_ %pred: -0.42 (men)  %LAA-950 and FEV_1_/FVC: -0.62 (men)  %LAA-950 and FEV_1_ %pred: -0.49 (women)  %LAA-950 and FEV_1_/FVC: -0.55 (women) | NA |
| Dransfleid et al, 2011 | MLD,%LAA<-950 | %LAA<-950 and FEV_1_%Pred: -0.215,%LAA<-950 and FEV_1_%Pred: -0.581 | %LAA<-950 and FEV_1_%Pred: -0.221,%LAA<-950 and FEV_1_%Pred: -0.612 |
| F. Mochizuki et al, 2019 | LAA% | LAA% and FEV_1_:-0.34  LAA% and FEV_1_/FVC:-0.36  LAA% and FEV_25-75_:-0.47  LAA% and FEV_25-75_/FVC:-0.56 |  |
| F. W. Feldhaus et al, 2019 | LAA% | LAA% and FEV_1_ %pred :-0.208 | LAA% and FEV_1_ %pred :-0.363 |
| Falaschi et al, 1995 [33] | %LAA-900, MLD, Visual Score | %LAA-900 and FEV_1_ %pred: -0.80  %LAA-900 and FEV_1_/FVC: -0.86  MLD and FEV_1_ %pred: 0.76  MLD and FEV_1_/FVC: 0.81 | %LAA-900 and FEV_1_ %pred: -0.82  %LAA-900 and FEV_1_/FVC: -0.86  MLD and FEV_1_ %pred: 0.85 MLD and FEV_1_/FVC: 0.89 |
| Fuseya et al, 2018 | %LAV-960 | %LAV and FEV_1_:-0.60 | No CT quantitative measurements |
| Gawlitza et al, 2018 | FWHM,LAV-950,MLD | FWHM and FEV_1_/FVC:0.1818 | No reports on the correlation to FEV_1_% Pred or FEV_1_/FVC |
| Gawlitza et al, 2018* | WA,LA,%WA，WT | WA and FEV_1_%Pred:0.151,LA and FEV_1_%Pred:0.029,%WA and FEV_1_% Pred:0.181,WT and FEV_1_%Pred:0.213 | WA and FEV_1_%Pred:0.269,LA and FEV_1_%Pred:0.205,%WA and FEV_1_% Pred: 0.02,WT and FEV_1_%Pred:0.245 |
| Gelb et al, 1993 [S11] | Visual Score | No CT quantitative measurements | NA |
| Grydeland et al, 2010 [S12] | %LAA-950, -910, etc. | No reports on the correlation to FEV_1_ %pred or FEV_1_/FVC | NA |
| Grydeland et al, 2011 [S13] | %LAA-950, WT | No reports on the correlation to FEV_1_ %pred or FEV_1_/FVC | NA |
| Haraguchi et al, 2016* | %LAA-950 | %LAA-950 and FEV_1_%Pred:-0.453 | NA |
| Hasegawa et al, 2006 [20]* | WA%, Ai, WA | WA% and FEV_1_ %pred: -0.547  Ai and FEV_1_ %pred: 0.731 | NA |
| Hesselbacher et al, 2011 [29]* | %LAA-950, etc. | %LAA-950 and FEV_1_/FVC: -0.71 (current smoker)  %LAA-950 and FEV_1_/FVC: -0.78 (former smoker) | NA |
| Heussel et al, 2009 [36] | %LAA-950, MLD, Perc15, LV, etc. | %LAA-950 and FEV_1_ %pred : -0.35  %LAA-950 and FEV_1_/FVC: -0.63  MLD and FEV_1_ %pred : 0.43  MLD and FEV_1_/FVC: 0.69  Perc15 and FEV_1_ %pred : 0.34  Perc15 and FEV_1_/FVC: 0.62  LV and FEV_1_ %pred : -0.02  LV and FEV_1_/FVC: -0.58 | NA |
| Hoshino et al, 2014* | %WA,Ai | %WA and FEV_1_:-0.442,Ai and FEV_1_:0.370 | NA |
| HUANG Xiao-qi et al, 2018 | MLD | MLD and FEV_1_ %pred :0.375  MLD and FEV_1_ /FVCpred :0.476 | MLD and FEV_1_ %pred :0.719  MLD and FEV_1_ /FVCpred :0.674 |
| Hyun Jung Koo et al, 2018 | Pi 10，MLD | MLD and FEV_1_ %pred :-0.20  Pi10 and FEV_1_ %pred :-3.14  Pi10 and FEV_1_ /FVC:-3.03 | NA |
| Iwasawa et al, 2007 [35] | %LAA-950, MLD | %LAA-950 and FEV_1_: -0.661  %LAA-950 and FEV_1_/FVC: -0.745  MLD and FEV_1_: 0.636  MLD and FEV_1_/FVC: 0.782 | NA |
| Iwasawa et al, 2011 [31]* | %LAA-950 | %LAA-950 and FEV_1_ %pred: -0.43  %LAA-950 and FEV_1_/FVC: -0.49 | NA |
| J. L. MacNeil, et al, 2020 | LAA% | LAA% and FEV_1_ %pred :-0.64  LAA% and FEV_1_ /FVC:-0.83 | NA |
| J.P. Charbonnier et al, 2019 | LAA% | NA | NA |
| Jin et al, 2007 [32] | %LAA-960, %LAA-950,  %LAA-910, %LAA-900, etc. | %LAA-960 and FEV_1_ %pred: -0.501  %LAA-960 and FEV_1_/FVC: -0.465  %LAA-950 and FEV_1_ %pred: -0.534  %LAA-950 and FEV_1_/FVC: -0.513  %LAA-910 and FEV_1_ %pred: -0.516  %LAA-910 and FEV_1_/FVC: -0.584  %LAA-900 and FEV_1_ %pred: -0.470  %LAA-900 and FEV_1_/FVC: -0.563 | %LAA-910 and FEV_1_ %pred: -0.562  %LAA-910 and FEV_1_/FVC: -0.506  %LAA-900 and FEV_1_ %pred: -0.571  %LAA-900 and FEV_1_/FVC: -0.523 |
| Jogi et al, 2011 [S14] | Emphysema percentage | No CT quantitative measurements | NA |
| Ju et al,2014* | %LAA-950 | %LAA-950 and FEV_1_%Pred: -1.54, %LAA-950 and FEV_1_/FVC:-1.09 | NA |
| Karayama et al,2017* | %LAA<950,WA,Ai | Ai and FEV_1_%Pred:0.392,Ai and FEV_1_/FVC:0.322,WT and FEV_1_% Pred:-0.189,WT and FEV_1_/FVC: -0.320,%LAA<950 and FEV_1_%Pred: -0.464,%LAA<950 and FEV_1_/FVC: -0.580 | No CT quantitative measurements |
| Kaya et al, 2017 | MLD | MLD and FEV_1_%Pred:-0.63,MLD and FEV_1_/FVC:-0.65 | MLD and FEV_1_%Pred:-0.89,MLD and FEV_1_/FVC:-0.88 |
| Kim et al, 2009 [S15] | %LAA-950, WT | %LAA-950 and FEV_1_ %pred: -0.07  WT and FEV_1_ %pred: -0.12 | NA |
| Kim et al, 2011 | %LAA-950,Pi10,Gas Trap | %LAA-950 and FEV_1_%Pred:-0.03,Gas Trap and FEV_1_%Pred:-0.69 | NA |
| Kim et al, 2013* | %LAA-950 ,%WA | %LAA-950 and FEV_1_%Pred: -0.62 %LAA-950 and FEV_1_/FVC: -0.60,%WA and FEV_1_%Pred: -0.551,%WA and FEV_1_/FVC:-0.495 | NA |
| Kim et al, 2015* | ATI,E/I,MLD,Exp-856 | ATI and FEV_1_:-0.725 ATI and FEV_1_/FVC:-0.737 MLD and FEV_1_: -0.715 MLD and FEV_1_/FVC: -0.730 | EI and FEV_1_:-0.568 EI and FEV_1_/FVC: -0.726 EXP-856 and FEV_1_:-0.724 EXP-856 and FEV_1_/FVC:-0.823 |
| Kim et al,2014 | MLD,EI | MLD and FEV_1_:0.3233,MLD and FEV_1_/FVC:0.4947,EI and FEV_1_: -0.4434,EI and FEV_1_/FVC:-0.5961 | NA |
| Kirby et al, 2017 | Gas Trap | PRM and FEV_1_%Pred:0.13 PRM and FEV_1_/FVC%:0.24 | PRM and FEV_1_%Pred:-0.15 PRM and FEV_1_/FVC%:-0.33 |
| Kosciuch et al, 2009 [11] | WA%, Ai, WA | No reports on the correlation with FEV_1_ %pred or FEV_1_/FVC in COPD subgroup | NA |
| Koyama et al, 2012 | %LAA-950,%LV, %WA | %LAA-950 and FEV_1_%Pred：-0.11，%LAA-950 and FEV_1_/FVC: -0.27,%LV and FEV_1_%Pred:0.53,%LV and FEV_1_/FVC:0.52,%WA and FEV_1_%Pred：-0.31，%WA and FEV_1_/FVC:-0.35 | NA |
| Kundu et al, 2013* | UL | UL and FEV_1_%Pred：0.492，UL and FEV_1_/FVC:0.493 | UL and FEV_1_%Pred：0.307，UL and FEV_1_/FVC:0.288 |
| Kuo-Lung Lor et al, 2019 | LAA% | LAA% and FEV_1_ %pred : -0.62  LAA% and FEV_1_ /FVC: -0.66 | NA |
| Kurashima et al, 2013* | %LAA,WA,Gas Trap | %LAA and FEV_1_%Pred:0.621 | NA |
| Lamers et al, 1994 [8] | Visual score | No CT quantitative measurements | No CT measurements |
| Lan Song et al, 2020 | TLV,TES,P_15_ | TLV and FEV_1_ %pred :-0.10  TLV and FEV_1_ /FVC:-0.49  TES and FEV_1_ %pred :-0.26  TES and FEV_1_ /FVC:-0.21  P_15_ and FEV_1_ %pred :0.23  P_15_ and FEV_1_ /FVC:0.23 | TLV and FEV_1_ %pred :-0.24  TLV and FEV_1_ /FVCpred :-0.43  TES and FEV_1_ %pred :-0.31  TES and FEV_1_ /FVCpred :-0.18  P_15_ and FEV_1_ %pred :0.33  P_15_ and FEV_1_ /FVCpred :0.18 |
| Leader et al, 2008 [21]* | WA%, Ai, Ao, WA | WA% and FEV_1_ %pred: -0.584  Ai and FEV_1_ %pred: 0.540  Ao and FEV_1_ %pred: 0.410  WA and FEV_1_ %pred: 0.172 | NA |
| Leader et al, 2009 [S16] | WA%, Ai, Ao, WA, etc. | WA% and FEV_1_ %pred: -0.238  WA% and FEV_1_/FVC: -0.180  Ai and FEV_1_ %pred: 0.286  Ai and FEV_1_/FVC: 0.237  Ao and FEV_1_ %pred: 0.149  Ao and FEV_1_/FVC: 0.128  WA and FEV_1_ %pred: -0.007  WA and FEV_1_ %pred: -0.001 | NA |
| Lee et al, 2008 [22]* | %LAA-950, MLD, Ai, WA, WA% | %LAA-950 and FEV_1_ %pred: -0.547  MLD and FEV_1_ %pred: 0.439  WA% and FEV_1_ %pred: -0.044 | %LAA-950 and FEV_1_ %pred: -0.553  MLD and FEV_1_ %pred: 0.619 |
| Lee et al, 2011 [S17] | %LAA-950 | No reports on the correlation with FEV_1_ %pred or FEV_1_/FVC | No reports on the correlation with FEV_1_ %pred or FEV_1_/FVC |
| Lee et al, 2011 [S18] | %LAA-950, MLD, LV, etc. | No reports on the correlation with FEV_1_ %pred or FEV_1_/FVC | No reports on the correlation with FEV_1_ %pred or FEV_1_/FVC |
| Lee et al, 2016* | ATI,E/I,MLD,Exp-856 | ATI and FEV_1_%Pred:-0.698,ATI and FEV_1_/FVC:-0.709,MLD and FEV_1_% Pred:-0.712,MLD and FEV_1_/FVC: -0.714 | %LAA-856 and FEV_1_%Pred: -0.612,%LAA-856 and FEV_1_/FVC:-0.64 |
| Li et al, 2009 [S19] | LV, etc. | LV and FEV_1_ %pred: 0.315  LV and FEV_1_/FVC: 0.191 | LV and FEV_1_ %pred: -0.616  LV and FEV_1_/FVC: -0.543 |
| Li et al, 2019 | Emph%,fSAD% | Emph% and FEV_1_%pred :-0.470  Emph% and FEV_1_ /FVC:-0.334  fSAD and FEV_1_%pred :-0.453  fSAD and FEV_1_ /FVC:-0.320 |  |
| Li Yan et al, 2020 | LAA%-950 | LAA% and FEV_1_%pred :-0.477  LAA% and FEV_1_ /FVC:-0.641 | NA |
| Li Yan et al, 2020 | LAA%-950ins,MLD | LAA%-950ins and FEV_1_%pred :-0.400  LAA%-950ins and FEV_1_ /FVC:-0.496 | NA |
| Madani et al, 2010 [S20] | %LAA-980 to -900(step 10%)  Perc1 to 18 (step 2-3%) | No reports on the correlation with FEV_1_ %pred or FEV_1_/FVC |  |
| Marquez-Martin et al , 2011 [S21] | Visual score | No CT quantitative measurements | NA |
| Matin et al,2017 | %LAA-950,Pi10 | %LAA-950 and FEV_1_%Pred: -0.20 %LAA-950 and FEV_1_/FVC: -0.22, %LAA-950 and Pi10: -0.24,%LAA-950 and Pi10:-0.040 | No CT quantitative measurements |
| Matsuoda et al, 2007 [14] | %LAA-950, %LAA-900 | %LAA-950 and FEV_1_ %pred: -0.471  %LAA-950 and FEV_1_/FVC: -0.428  %LAA-900 and FEV_1_ %pred: -0.404  %LAA-900 and FEV_1_/FVC: -0.320 | %LAA-950 and FEV_1_ %pred: -0.602  %LAA-950 and FEV_1_/FVC: -0.554  %LAA-900 and FEV_1_ %pred: -0.618  %LAA-900 and FEV_1_/FVC: -0.525 |
| Matsuoda et al, 2008 [S22] | Ai, etc. | Ai and FEV_1_ %pred: 0.26  Ai and FEV_1_/FVC: 0.28 | Ai and FEV_1_ %pred: 0.63  Ai and FEV_1_/FVC: 0.64 |
| Matsuoda et al, 2008 [S23] | Percentage between two thresholds | No CT quantitative measurements | No CT quantitative measurements |
| Mets et al, 2011 [S24] | log%LAA-950, Perc15,etc. | log%LAA-950 and logFEV_1_: 0.53^†^  log%LAA-950 and FEV_1_/FVC: 0.61^†^  Perc15 and logFEV_1_: 0.44^†^  Perc15 and FEV_1_/FVC: 0.53^†^ | NA |
| Mishima et al, 1999 [S25] | %LAA-960 | %LAA-960 and FEV_1_ %pred: -0.320  %LAA-960 and FEV_1_/FVC: -0.528 | NA |
| Mohamed Hoesein, 2011 [45] | %LAA-950, Perc15 | %LAA-950 and FEV_1_ %pred: -0.16  %LAA-950 and FEV_1_/FVC: -0.42  Perc15 and FEV_1_ %pred: 0.12  Perc15 and FEV_1_/FVC: 0.39 | NA |
| Moron et al, 2004 [S26] | Visual score | No CT quantitative measurements | NA |
| Moroni et al, 2001 [S27] | %LAA-910, MLD | No reports on the correlation with FEV_1_ %pred or FEV_1_/FVC | %LAA-910 and FEV_1_/FVC: -0.78  MLD and FEV_1_/FVC: 0.85 |
| Myung HC et al, 2018 | LV | NA | NA |
| Nakano et al, 1999 [S28] | %LAA-960 | %LAA-960 and FEV_1_: -0.492  %LAA-960 and FEV_1_/FVC: -0.622 | NA |
| Nakano et al, 2000 [3] | %LAA-960  WA%, Ai, Ao, T/D ratio, etc. | %LAA-960 and FEV_1_ %pred: -0.529  %LAA-960 and FEV_1_/FVC: -0.650  WA% and FEV_1_ %pred: -0.338  Ai and FEV_1_ %pred: 0.273  Ao and FEV_1_ %pred: 0.195  WA% and FEV_1_/FVC -0.192 | NA |
| Nambu et al, 2015 | %LAA-856,Perc15 | %LAA-856 and FEV_1_%Pred: 0.112,%LAA-856 and FEV_1_/FVC:0.258 Perc15 and FEV_1_% Pred: 0.218,Perc15 and FEV_1_/FVC:0.392 | %LAA-856 and FEV_1_%Pred: 0.518,%LAA-856 and FEV_1_/FVC:0.634 |
| Nishio et al, 2016* | %LAA,D | %LAA and FEV_1_:-0.565,D and FEV_1_:0.0934 | NA |
| Nishio M et al, 2018 | LAV%,CSA%,WA% | LAV% and FEV_1_ :-0.505  LAV% and FEV_1_ /FVC:-0.640  CSA% and FEV_1_ :0.384  CSA% and FEV_1_ /FVC:0.288  WA% and FEV_1_ :-0.196  WA% and FEV_1_ /FVC:-0.131 | NA |
| O. Doganay et al, 2018 | LAA% | LAA% and FEV_1_ %pred :-0.635  LAA% and FEV_1_ /FVC:-0.652 |  |
| Occhipinti et al, 2018* | %LAA-950,%LAA-856,%DLCO | %LAA-950 and FEV_1_/FVC: -0.66, %LAA-950 and %DLCO:-0.43 | %LAA-856 and FEV_1_/FVC : -0.64 ,%LAA-856 and %DLCO:-0.43 |
| Occhipinti et al, 2019 | LAA%-950ins,LAA%-856exp,WT,fLDA%,pLDA% | LAA%-950ins and FEV_1_%pred :-0.50  LAA%-950ins and FEV_1_ /FVC:-0.67  WT and FEV_1_%pred :-0.22  WT and FEV_1_ /FVC:-0.11  fLDA% and FEV_1_%pred :-0.42  fLDA% and FEV_1_ /FVC:-0.49  pLDA% and FEV_1_%pred :-0.52  pLDA% and FEV_1_ /FVC:-0.68 | LAA%-856exp and FEV_1_%pred :-0.58  LAA%-856exp and FEV_1_ /FVC:-0.71 |
| O'Donnel et al, 2004 [40] | %LAA-950, MLD | %LAA-950 and FEV_1_ %pred: -0.45  MLD and FEV_1_ %pred: 0.38 | %LAA-950 and FEV_1_ %pred: -0.52  MLD and FEV_1_ %pred: 0.63 |
| Oelsner et al, 2016 | %LAA<-950,%HAA（-250-600） | %LAA<-950 and FEV_1_/FVC: -0.37,%HAA and FEV_1_/FVC:-0.14 | NA |
| Oh,S.Y et al, 2017* | %LAA-950 | %LAA-950 and FEV_1_%Pred: -0.533 %LAA-950 and FEV_1_/FVC: -0.663 | %LAA-950 and FEV_1_%Pred: -0.567,%LAA-950 and FEV_1_/FVC:-0.660 |
| Ohara et al, 2006 [S29] | %LAA-960, WA%, Ai, Ao, T/D ratio, etc. | Separately reported by lung fields  %LAA-960 and FEV_1_ %pred: -0.331 (upper)  %LAA-960 and FEV_1_/FVC: -0.222 (upper)  %LAA-960 and FEV_1_ %pred: -0.487 (lower)  %LAA-960 and FEV_1_/FVC: -0.491 (lower)  WA% and FEV_1_ %pred: -0.336 (upper)  WA% and FEV_1_/FVC: -0.280 (upper)  WA% and FEV_1_ %pred: -0.339 (lower)  WA% and FEV_1_/FVC: -0.357 (lower) | NA |
| Ohno et al, 2011 [30]* | WA%, etc. | WA% and FEV_1_ %pred: -0.69  WA% and FEV_1_/FVC: -0.59 | NA |
| Ohno et al, 2012* | FLA,%WA,WT | FLV and FEV_1_%Pred:0.64,FLV and FEV_1_/FVC:-0.58,%WA and FEV_1_% Pred:-0.69%,%WA and FEV_1_/FVC: -0.59,WT and FEV_1_%Pred:-0.68,WT and FEV_1_/FVC:-0.62 | NA |
| Orlandi et al, 2004 [15] | %LAA-950, -910, MLD | %LAA-950 and FEV_1_ %pred: -0.59  %LAA-950 and FEV_1_/FVC: -0.65  Separately reported by radiation dose  %LAA-910 and FEV_1_ %pred: -0.91 (normal)  %LAA-910 and FEV_1_/FVC: -0.81 (normal)  MLD and FEV_1_ %pred: 0.77 (normal)  MLD and FEV_1_/FVC: 0.78 (normal)  %LAA-910 and FEV_1_ %pred: -0.87 (low)  %LAA-910 and FEV_1_/FVC: -0.81 (low)  MLD and FEV_1_ %pred: 0.57 (low)  MLD and FEV_1_/FVC: -0.36 (low) | NA |
| Orlandi et al, 2005 [S30] | %LAA-950, MLD, WA%, WA, T/D ratio | %LAA-950 and FEV_1_ %pred: -0.42  %LAA-950 and FEV_1_/FVC: -0.50  MLD and FEV_1_ %pred: 0.40  MLD and FEV_1_/FVC: 0.59  WA% and FEV_1_ %pred: -0.04  WA% and FEV_1_/FVC: -0.009 | NA |
| Paoletti et al, 2015 | %LAA-950,%LAA-910 | %LAA-950 and FEV_1_%Pred: -0.44,%LAA-950 and FEV_1_/FVC:-0.53 | %LAA-910 and FEV_1_% Pred: -0.67,%LAA-910 and FEV_1_/FVC:-0.72 |
| Park et al, 2008 [23]* | %LAA-950 | %LAA-950 and FEV_1_ %pred: -0.46  %LAA-950 and FEV_1_/FVC: -0.67 | NA |
| Patel et al, 2008 [2] | %LAA-950, %LAA-910,  Visual score | %LAA-950 and FEV_1_ %pred: -0.31  %LAA-950 and FEV_1_/FVC: -0.41  No reports on the correlation between %LAA-910 and FEV_1_ %pred or FEV_1_/FVC | NA |
| Pauls et al, 2010 [24]* | %LAA-950, LV | %LAA-950 and FEV_1_ %pred: -0.360  LV and FEV_1_ %pred: -0.162 | NA |
| Pescarolo et al, 2008 [S31] | Visual score | No CT quantitative measurements | NA |
| Petersen et al, 2010 [S32] | WA%, Ai, Ao | No reports on the correlation with FEV_1_ %pred or FEV_1_/FVC in COPD patients | NA |
| Sandek et al, 2002 [S33] | %LAA-910, MLD | %LAA-910 and FEV_1_ %pred: -0.69  %LAA-910 and FEV_1_/FVC: -0.76  MLD and FEV_1_ %pred: 0.36  MLD and FEV_1_/FVC: 0.54 | %LAA-910 and FEV_1_ %pred: -0.83  %LAA-910 and FEV_1_/FVC: -0.87 |
| Saruya et al, 2016* | %LAA<-950,%CSA | %CSA and FEV_1_%Pred: 0.53,%LAA<-950 and FEV_1_% Pred:-0.41 | NA |
| Sasaki et al, 2014* | LA,%WA | LA and FEV_1_%Pred:0.443,LA and FEV_1_/FVC:0.512,%WA and FEV_1_% Pred:-0.499,%WA and FEV_1_/FVC: -0.515 | NA |
| Scichilone et al, 2008 [S34] | MLD, etc | No reports on the correlation between MLD and FEV_1_ %pred or FEV_1_/FVC | NA |
| Shah et al, 2020 | Tracheal index | Tracheal index and FEV_1_:0.419  Tracheal index and FEV_1_ /FVC:0.552  Tracheal index and RV/TLC:-0.530 | NA |
| Shaikh et al, 2017 | %LAA-950,%LAA-856,%DLCO | %LAA -950and FEV_1_%Pred :-0.589 | No CT quantitative measurements |
| Shaker et al, 2005 [37] | %LAA-910, Perc15, etc. | %LAA-910 and FEV_1_ %pred: -0.62  %LAA-910 and FEV_1_/FVC: -0.62  Perc15 and FEV_1_ %pred: 0.62  Perc15 and FEV_1_/FVC: 0.61 | NA |
| Shuyi Qin et al, 2021 | WA/AA, LA/AA | WA/AA_3_ and FEV_1_ %pred :-0.428  WA/AA_5_ and FEV_1_ %pred :-0.532  WA/AA_9_ and FEV_1_ %pred :-0.570  LA/AA_3_ and MMEF%:0.421  LA/AA_5_ and MMEF%:0.566  LA/AA_9_ and MMEF%:0.610  WA/AA_3_ and MMEF%:-0.421  WA/AA_5_ and MMEF%:-0.529  WA/AA_9_ and MMEF%:-0.593 | NA |
| Sileikiene et al, 2017* | %LAV-950 | %LAV and FEV_1_%Pred:-0.505, %LAV and FEV_1_/FVC:-0.640 | No CT quantitative measurements |
| Sorensen et al, 2010 [S35] | Emphysema pattern | No CT quantitative measurements | NA |
| Spiropoulos et al, 2003 [S36] | %LAA-910 | No reports on the correlation with FEV_1_ %pred or FEV_1_/FVC | No reports on the correlation to FEV_1_ %pred or FEV_1_/FVC |
| Suzuki et al, 2020 | LAA%,HAA%,AA% | LAA% and FEV_1_%pred :-0.29  LAA% and FEV_1_ /FVC:-0.23  HAA% and FEV_1_%pred :-0.29  HAA% and FEV_1_ /FVC:0.29  AA% and FEV_1_%pred :-0.45  AA% and FEV_1_ /FVC:0.13 | NA |
| T. Xia et al, 2020 | Inspiratory VI-950  Expiratory VI-856  MLD_E/I_ | NA | NA |
| Timmins et al, 2012* | %LAA<910 | %LAA<910 and FEV_1_%Pred: -0.28,%LAA<910 and FEV_1_/FVC:-0.57 | NA |
| Torres et al, 2011 [S37] | %LAA-960 | %LAA-960 and FEV_1_ %pred: 0.14  %LAA-960 and FEV_1_/FVC: -0.24 | NA |
| Tsushima et al, 2010 [S38] | %LAA-960, Visual score | %LAA-960 and FEV_1_/FVC: -0.29 | NA |
| Van Der Lee et al, 2006 [S39] | %LAA-950, LV | %LAA-950 and FEV_1_ %pred: 0.3^†^  No reports on the correlation between LV and FEV_1_ %pred or FEV_1_/FVC | NA |
| Wang et al, 2015* | %LAV-950 | %LAV-950 and FEV_1_% Pred: -0.67,%LAV-950 and FEV_1_/FVC:-0.68 | NA |
| Washko et al, 2008 [12] | %LAA-950, -910, MLD, Perc15, etc | %LAA-950 and FEV_1_ %pred: -0.09  %LAA-950 and FEV_1_/FVC: -0.09  %LAA-910 and FEV_1_ %pred: -0.20  %LAA-910 and FEV_1_/FVC: -0.19  MLD and FEV_1_ %pred: 0.18  MLD and FEV_1_/FVC: 0.21  Perc15 and FEV_1_ %pred: 0.09  Perc15 and FEV_1_/FVC: 0.12 | NA |
| Washko et al, 2009 [25]* | %LAA-950, WA%, Ai, WT | WA% and FEV_1_ %pred: -0.28  WA% and FEV_1_/FVC: -0.014  Ai and FEV_1_ %pred: 0.14  Ai and FEV_1_FVC: 0.07  WT and FEV_1_ %pred: -0.13  WT and FEV_1_/FVC: -0.05 | NA |
| Washko et al, 2014 | WA,Ai,Ao,%WA,Ao | WA and FEV_1_%Pred:0.15,Ai and FEV_1_%Pred:0.18,Ao and FEV_1_% Pred:0.17,%WA and FEV_1_%Pred:-0.26 | NA |
| Watanuki et al, 1994 [S40] | MLD | MLD and FEV_1_ %pred: 0.72 | NA |
| Xie et al,2016 | %LAA-950,%LAA-910,Perc15 | No reports on the correlation to FEV_1_%pred or FEV_1_/FVC | No reports on the correlation to FEV_1_%pred or FEV_1_/FVC |
| Yahaba et al,2014* | Ai,%WA | Ai and FEV_1_:0.50,%WA and FEV_1_:-0.53 | Ai and FEV_1_:0.34,%WA and FEV_1_:-0.40 |
| Yamashiro et al, 2010 [26]* | %LAA-950, MLD, LV, etc. | %LAA-950 and FEV_1_ %pred: -0.625  %LAA-950 and FEV_1_/FVC: -0.713  MLD and FEV_1_.%pred: 0.494  MLD and FEV_1_/FVC: 0.562  LV and FEV_1_ %pred: -0.010  LV and FEV_1_/FVC: -0.198 | %LAA-950 and FEV_1_ %pred: -0.637  %LAA-950 and FEV_1_/FVC: -0.729  MLD and FEV_1_.%pred: 0.661  MLD and FEV_1_/FVC: 0.743  LV and FEV_1_ %pred: -0.406  LV and FEV_1_/FVC: -0.588 |
| Yamashiro et al, 2010 [28]* | %LAA-950, WA%, Ai, etc. | %LAA-950 and FEV_1_ %pred: -0.460  WA% and FEV_1_ %pred: -0.470  Ai and FEV_1_ %pred: 0.450 | NA |
| Yamashiro et al, 2011 [S41] | Kurtosis and skewness | No CT quantitative measurements | No CT quantitative measurements |
| Zampatori et al, 1997 [S42] | %LAA-900, Visual score | No reports on the correlation with FEV_1_ %pred or FEV_1_/FVC | %LAA-900 and FEV_1_ %pred: -0.65 |
| Zampatori et al, 2001 [S43] | Visual score | No CT measurements | NA |
| Zampatori et al, 2001 [S44] | %LAA-900, LV, Visual score | LV and FEV_1_ %pred: -0.49  LV and FEV_1_/FVC: -0.69 | NA |
| Zampatori et al, 2002 [S45] | %LAA-900, MLD, LV,  Visual score | %LAA-900 and FEV_1_ %pred: -0.53 (Scan 1)  %LAA-900 and FEV_1_ %pred: -0.56(Scan 2)  %LAA-900 and FEV_1_/FVC: -0.79 (Scan 1)  %LAA-900 and FEV_1_/FVC: -0.80 (Scan 2)  No reports on the correlation between MLD, LV and FEV_1_ %pred or FEV_1_/FVC | NA |
| Zaporozhan et al, 2005 [41] | %-LAA-950, MLD, LV, etc | No reports on the correlation with FEV_1_ %pred or FEV_1_/FVC | No reports on the correlation to FEV_1_ %pred or FEV_1_/FVC |
| Zhang D et al, 2018 | LAA%-950,P15-_IN_,P15-_EX_,E/I _MLD_ | LAA% and FEV_1_ %pred :0.10  LAA% and FEV_1_ /FVC:-0.21  LAA% and RV/TLC:0.59  LAA% and DLCO/VA:-0.62  P15-_IN_ and FEV_1_ %pred :-0.29  P15-_IN_ and FEV_1_ /FVC :0.26  P15-_IN_ and RV/TLC:-0.40  P15-_IN_ and DLCO/VA:0.53  E/I _MLD_ and FEV_1_ %pred :-0.00  E/I _MLD_ and FEV_1_ /FVC :-0.19  E/I _MLD_ and RV/TLC:0.06  E/I _MLD_ and DLCO/VA:-0.19 | P15-_EX_ and FEV_1_ %pred :-0.22  P15-_EX_ and FEV_1_ /FVCpred :0.33  P15-_EX_ and RV/TLC:-0.23  P15-_EX_ and DLCO/VA:0.55 |
| Zhang et al, 2008 [27]* | %-LAA-950, -910, MLD | %LAA-950 and FEV_1_ %pred: -0.520  %LAA-950 and FEV_1_/FVC: -0.626  MLD and FEV_1_ %pred: 0.416  MLD and FEV_1_/FVC: 0.512  %LAA-910 and FEV_1_ %pred: -0.437  %LAA-910 and FEV_1_/FVC: -0.548 | NA |
| Zhang et al, 2015* | %LAA-950,%WA | %LAA-950 and FEV_1_%Pred: -0.65, %LAA-950 and FEV_1_/FVC : -0.6,%WA and FEV_1_% Pred: -0.54,%WA and FEV_1_/FVC: -0.45 | NA |
| Zhao et al, 2019 | LAA%-950ins,LAA%-856exp,Emph% | LAA%-950ins and FEV_1_%pred :-0.272  LAA%-950ins and FEV_1_ /FVC:-0.458  LAA%-950ins and DLCO:-0.347  LAA%-950ins and RV/TLC:0.230  Emph% and FEV_1_ /FVC:-0.358  Emph% and DLCO:=-0.422  Emph% and RV/TLC:0.342 | LAA%-856exp and FEV_1_%pred :-0.210  LAA%-856exp and FEV_1_ /FVC:-0.276  LAA%-856exp and DLCO:0.222  LAA%-856exp and RV/TLC:0.159 |

NA = Not available; %LAA = Percentage low attenuation area; MLD = Mean lung density; LV = Lung volume; Perc = Percentile point of lung density; WA% = Airway wall area percentage; Ai = Airway lumen area; Ao = Total airway area; WT = Wall thickness; T/D radio = Ratio of airway wall thickness to total diameter.

* Included in the meta-analysis.
