# Supplementary material for: Quantitative CT parameters correlate with lung function in chronic obstructive pulmonary disease: A systematic review and meta-analysis
Source: Front Surg. 2023 Jan 4;9:1066031. doi: 10.3389/fsurg.2022.1066031 (PMC9845891; doi:10.3389/fsurg.2022.1066031)
Supplement: Supplementary Table S4 — Characteristics of studies included in the meta-analysis [file Table5.docx]

**Electronic supplementary table 5 Characteristics of studies included in the meta analyse**

| **Study, Year** | **Patients, *n*** | **Men, %** | **Age,**  **Year ± SD or**  **year (range)** | **COPD Severity,**  **GOLD stage or**  **FEV_1_ %pred ± SD or FEV_1_ %pred (range)** | **CT type, slice** | **Inspiratory or expiratory CT examination** | **Volumetric CT examination** | **Radiation Dose** | **Cohort Name** |
| --- | --- | --- | --- | --- | --- | --- | --- | --- | --- |
| Abd et al,2020 | 50 | 100 | 62.82±8.65 | Stage0-IV | 16 | Inspiratory | Volume | Normal |  |
| Akira et al, 2009 [13]* | 76 | 88 | 67(37-85) | Stage 0-IV | 16 | Both | Volume | Normal |  |
| Bon et al, 2009 [18]* | 234 | 50 | 61(50-78) | Stage 0-IV | 4&8 | Inspiratory | Volume | Low | PLuSS |
| Capaldi et al,2016* | 58 | 69 | 73±9 | Stage I-IV | 64 | Both | Volume | Low |  |
| Dransfield et al, 2007 [19]* | 396 | 62 | 63±5(*n*=246)  61±5(*n*=150) | Stage 0-IV | Multi | Inspiratory | Volume | Low | NLST |
| Feldhaus et al,2019 | 88 | 60 | 66±6.6 (45-79) | StageIII-IV | NA | Both | Volume | Normal |  |
| Gawlitza et al,2018* | 65 | NA | NA | Stage I-IV | 4 | Both | Volume | Low |  |
| Haraguchi et al,2016* | 443 | 92 | 72.6±8.2 | Stage I-IV | Multi | Inspiratory | Volume | Low |  |
| Hasegawa et al, 2006 [20]* | 52 | 96 | 72(41-84) | Stage I-IV | 4 | Inspiratory | Volume | Normal | Hokkaido |
| Hesselbacher et al, 2011 [29]* | 224 | 65 | >40 | Stage I-IV | 64 | Inspiratory | Volume | Normal | LESCOPD |
| Hoshino et al,2014* | 54 | NA | >40 | Stage I-IV | 64 | Inspiratory | Non-volume | Normal |  |
| HUANG et al,2018 | 60 | 78 | 47-78 | StageI-IV | 128 | Both | Volume | Normal |  |
| Hyun Jung Koo et al,2018 | 370 | 97 | 65-75 | StageI-IV | 16&64 | Both | Volume | Normal |  |
| Iwasawa et al, 2011 [31]* | 35 | 100 | 70±6 | Stage I-IV | 16 | Inspiratory | Volume | Normal |  |
| Ju et al,2014* | 350 | 57 | 64 | Stage 0-IV | 64 | Inspiratory | Non-volume | Low |  |
| Karayama et al,2017* | 147 | 93 | 73 | Stage I-IV | 64 | Inspiratory | Volume | Low |  |
| Kim et al,2013* | 200 | 64 | 64.6±8.4 | Stage I-IV | 16&64 | Inspiratory | Volume | Low |  |
| Kim et al,2015* | 167 | 89 | 64.8±8.2(n=138) 51.7±9.4(n=29) | Stage 0-IV | 16 | Both | Non-volume | Low |  |
| Kundu et al,2013* | 180 | 52 | 65.6±9.7 | Stage 0-IV | 16 | Both | Non-volume | Normal |  |
| Kuo-Lung Lor et al, 2019 | 87 | 97 | 67.3 | StageI-IV | 4 | Inspiratory | Volume | Normal |  |
| Kurashima et al,2013* | 85 | 100 | 70.8±6.7 | Stage I-IV | Multi | Inspiratory | Volume | Low |  |
| Leader et al, 2008 [21]* | 240 | NA | NA | Stage 0-IV | 4(*n*=112)  8(*n*=128) | Inspiratory | Volume | Low | PLuSS |
| Lee et al, 2008 [22]* | 34 | 97 | 65(50-78) | 45%(17-82%) | 16 | Both | Volume | Normal | KOLD |
| Lee et al,2016* | 174 | 91 | 62.6±9.8 | Stage 0-IV | 16 | Both | Volume | Low | KOLD |
| Li Yan et al,2020 | 248 | NA | 47-79 | StageI-IV | 64 | Inspiratory | Volume | Normal |  |
| Li Yan et al,2020 | 32 | 84 | 65.8±10.55 | StageII-III | 64 | Inspiratory | Volume | Normal |  |
| MacNeil, et al,2020 | 175 | 61.7 | 69±9 | Stage0-IV | 64 | Inspiratory | Volume | Normal |  |
| Mochizuki et al,2019 | 133 | 74.4 | 70(61-75) | StageI-III | NA | Inspiratory | Volume | Normal |  |
| Nishio et al,2016* | 30 | 83 | 70.1±12.1 | Stage I-IV | 16 | Inspiratory | Volume | Low |  |
| Nishio M et al,2018 | 87 | 77 | 67.4±11.0 | StageI-III | 320 | Inspiratory | Volume | Normal |  |
| Occhipinti et al,2018* | 202 | 78 | 70.3±8.1 | Stage I-IV | 64 | Both | Volume | Both |  |
| Occhipinti et al,2019 | 194 | 79 | 70±8 | StageII-III | 64&128 | Both | Volume | Normal |  |
| Oh,S.Y et al,2017* | 72 | 91 | 63.6(45-79) | Stage II-IV | 16 | Both | Volume | Low |  |
| Ohno et al, 2011 [30]* | 186 | 65 | (23-87) | Stage 0-IV | 16&64 | Inspiratory | Volume | Normal |  |
| Ohno et al,2012* | 187 | 6.5 | 23-87 | Stage 0-IV | 16&64 | Inspiratory | Volume | Low |  |
| Park et al, 2008 [23]* | 39 | 92 | 66(51-79) | 44±15% | 16 | Inspiratory | Volume | Normal | KOLD |
| Pauls et al, 2010 [24]* | 145 | NA | NA | Stage I-IV | 16 | Inspiratory | Volume | Normal |  |
| Saruya et al,2016* | 74 | 85 | 73(47-90) | Stage I-IV | 64 | Inspiratory | Volume | Low |  |
| Sasaki et al,2014* | 32 | 84 | 70.0±6.8 | Stage 0-IV | 16 | Inspiratory | Non-volume | Normal |  |
| Sileikiene et al,2017* | 59 | 88 | 68.36±11.13 | Stage I-IV | 64 | Inspiratory | Volume | Low |  |
| Suzuki et al,2020 | 46 | 97.8 | 67.2±7.8 | Stage0-I | 64 | Inspiratory | Volume | Normal |  |
| Timmins et al,2012* | 26 | 61 | 69.6±8.0 | Stage I-III | 4 | Both | Volume | Low |  |
| Wang et al,2015* | 46 | 80 | 67.0±10.84 | Stage I-IV | 64&128 | Inspiratory | Volume | Low |  |
| Washko et al, 2009 [25]* | 224 | 42 | 62±5 | Stage I-IV | 4 | Inspiratory | Volume | Low | NLST |
| Yahaba et al,2014* | 91 | NA | 68.9±7.9 | Stage I-IV | 64 | Both | Non-volume | Normal |  |
| Yamashiro et al, 2010 [26]* | 46 | 57 | 68(46-81) | Stage 0-IV | 16 | Both | Volume | Normal | LTRC |
| Yamashiro et al, 2010 [28]* | 114 | 57 | 62(56-74) | Stage I-IV | 4 | Inspiratory | Volume | Low | NLST |
| Zhang D et al,2018 | 24 | 62.5 | 62±8(48-77) | StageI-II | 256 | Both | Volume | Normal |  |
| Zhang et al, 2008 [27]* | 50 | 66 | 67±10 | Stage 0-IV | 16 | Inspiratory | Volume | Normal |  |
| Zhang et al,2015* | 112 | 60 | 66.97±10.46 | Stage 0-IV | 64 | Inspiratory | Volume | Low |  |
| Zhao et al,2019 | 87 | 71.2 | 46.35±6.56 | StageI-III | 128 | Both | Volume | Normal |  |

SD = Standard deviation; SE = Standard error; GOLD = The Global Initiative for Chronic Obstructive Lung Disease; FEV_1_ %pred = Predicted forced expiratory volume in the first second; PFT = Pulmonary function test; NA = Not available; VC = Vital capacity; BCOPDE = Biomarkers in COPD exacerbation; PLuSS = the Pittsburgh Lung Screening Study; NLST = the National Lung Screening Trial; GenKOLS = the Genetic COPD Study; LES-COPD = Longitudinal Exacerbation Study of COPD; NETT = the National Emphysema Treatment Trial; KOLD = the Korean Obstructive Lung Disease; NELSON = the Dutch-Belgian Lung Cancer Screening Trial; ICGN = the International COPD Genetics Network; DLCST = the Danish Lung Cancer Screening Trial; LTRC = the National Heart, Lung and Blood Institute Lung Tissue Research Consortium.

* Included in the meta-analysis.

† Expressed as mean ± standard error.
