# Supplementary material for: Quantitative CT parameters correlate with lung function in chronic obstructive pulmonary disease: A systematic review and meta-analysis
Source: Front Surg. 2023 Jan 4;9:1066031. doi: 10.3389/fsurg.2022.1066031 (PMC9845891; doi:10.3389/fsurg.2022.1066031)
Supplement: Supplementary Table S5 — Correlation coefficients between CT measurements and airflow obstruction parameters of pulmonary function test in the meta-analysis [file Table6.docx]

**Electronic supplementary table 6 Correlation coefficients between CT measure -ments and airflow obstruction parameters of pulmonary function test in the meta analysis**

| **Study, Year** | **CT Measurements** | **Correlation coefficients between CT measurements and airflow obstruction, *r*** | |
| --- | --- | --- | --- |
|  |  | **Inspiratory scan** | **Expiratory scan** |
| Abd et al, 2020 | LAA% | LAA% and FEV_1_:-0.313  LAA% and FVC:-0.272  LAA% and FEV_1_/FVC:-0.413 | NA |
| Akira et al, 2009 [13]* | %LAA-950, -910, MLD, Perc15 Visual score, etc. | %LAA-950 and FEV_1_ %pred: -0.659  %LAA-950 and FEV_1_/FVC: -0.712  %LAA-910 and FEV_1_ %pred: -0.601  %LAA-910 and FEV_1_/FVC: -0.661  MLD and FEV_1_ %pred: 0.694  MLD and FEV_1_/FVC: 0.764  Perc15 and FEV_1_ %pred: 0.292  Perc15 and FEV_1_/FVC: 0.600 | %LAA-950 and FEV_1_ %pred: -0.668  %LAA-950 and FEV_1_/FVC: -0.666  %LAA-910 and FEV_1_ %pred: -0.632  %LAA-910 and FEV_1_/FVC: -0.642  MLD and FEV_1_ %pred: 0.792  MLD and FEV_1_/FVC: 0.721  Perc15 and FEV_1_ %pred: 0.352  Perc15 and FEV_1_/FVC: 0.544 |
| Bon et al, 2009 [18]* | %LAA-950, WA% | %LAA-950 and FEV_1_ %pred: -0.43  WA% and FEV_1_ %pred : -0.39 | NA |
| Capaldi et al, 2016* | ATI,%LAA-950 | ATI and FEV_1_%Pred:-0.29,ATI and FEV_1_/FVC:-0.58,%LAA-950 and FEV_1_%Pred:-0.43,%LAA-950 and FEV_1_/FVC:-0.52 | No reports on the correlation to FEV1%pred or FEV1/FVC |
| Dransfield et al, 2007 [19]* | %LAA-950 | %LAA-950 and FEV_1_ %pred: -0.44 (total)  %LAA-950 and FEV_1_/FVC: -0.58 (total)  %LAA-950 and FEV_1_ %pred: -0.42 (men)  %LAA-950 and FEV_1_/FVC: -0.62 (men)  %LAA-950 and FEV_1_ %pred: -0.49 (women)  %LAA-950 and FEV_1_/FVC: -0.55 (women) | NA |
| Feldhaus et al, 2019* | LAA% | LAA% and FEV_1_ %pred : -0.208 | LAA% and FEV_1_ %pred : -0.363 |
| Gawlitza et al, 2018* | WA,LA,%WA，WT | WA and FEV_1_:0.151  LA and FEV_1_:0.029  %WA and FEV_1_:0.181  WT and FEV_1_:0.213 | WA and FEV_1_:0.269,LA and FEV_1_:0.205,%WA and FEV_1_: 0.02,WT and FEV_1_:0.245 |
| Haraguchi et al, 2016* | %LAA-950 | %LAA-950 and FEV_1_%Pred:-0.453 | NA |
| Hasegawa et al, 2006 [20]* | WA%, Ai, WA | WA% and FEV_1_ %pred: -0.547  Ai and FEV_1_ %pred: 0.731 | NA |
| Hesselbacher et al, 2011 [29]* | %LAA-950, etc. | %LAA-950 and FEV_1_/FVC: -0.71 (current smoker)  %LAA-950 and FEV_1_/FVC: -0.78 (former smoker) | NA |
| Hoshino et al, 2014* | %WA,Ai | %WA and FEV_1_:-0.442, Ai and FEV_1_: 0.370 | NA |
| HUANG et al, 2018 | MLD | MLD and FEV_1_ %pred :0.375  MLD and FEV_1_ /FVC:0.476 | MLD and FEV_1_ %pred :0.719  MLD and FEV_1_ /FVC :0.674 |
| Hyun Jung Koo et al, 2018 | Pi 10，MLD | MLD and FEV_1_ %pred :-0.20  Pi10 and FEV_1_ %pred :-3.14  Pi10 and FEV_1_ /FVC:-3.03 | NA |
| Iwasawa et al, 2011 [31]* | %LAA-950 | %LAA-950 and FEV_1_ %pred: -0.43  %LAA-950 and FEV_1_/FVC: -0.49 | NA |
| Ju et al, 2014* | %LAA-950 | %LAA-950 and FEV_1_%Pred: -1.54  %LAA-950 and FEV_1_/FVC:-1.09 | NA |
| Karayama et ai, 2017* | %LAA<950,WT,Ai | Ai and FEV_1_%Pred:0.392  Ai and FEV_1_/FVC:0.322  WT and FEV_1_% Pred:-0.189  WT and FEV_1_/FVC: -0.320  %LAA<950 and FEV_1_%Pred: -0.464  %LAA<950 and FEV_1_/FVC: -0.580 | No CT quantitative measurements |
| Kim et al, 2013* | %LAA-950 ,%WA | %LAA-950 and FEV_1_%Pred: -0.62  %LAA-950 and FEV_1_/FVC: -0.60  %WA and FEV_1_%Pred: -0.551  %WA and FEV_1_/FVC:-0.495 | NA |
| Kim et al, 2015* | ATI,E/I,MLD,Exp-856 | ATI and FEV_1_:-0.725  ATI and FEV_1_/FVC:-0.737  MLD and FEV_1_: -0.715  MLD and FEV_1_/FVC: -0.730 | EI and FEV_1_:-0.568 EI and FEV_1_/FVC: -0.726 EXP-856 and FEV_1_:-0.724 EXP-856 and FEV_1_/FVC:-0.823 |
| Kundu et al, 2013* | UL | UL and FEV_1_%Pred: 0.49  UL and FEV_1_/FVC: 0.493 | UL and FEV_1_%Pred：0.307，UL and FEV_1_/FVC:0.288 |
| Kuo-Lung Lor et al, 2019* | LAA% | LAA% and FEV_1_ %pred : -0.62  LAA% and FEV_1_ /FVC: -0.66 | NA |
| Kurashima et al, 2013* | %LAA,WA,ATI | %LAA and FEV_1_%Pred:0.621 | NA |
| Leader et al, 2008 [21]* | WA%,Ai,Ao,WA,etc | WA% and FEV_1_ %pred: -0.238  WA% and FEV_1_/FVC: -0.180  Ai and FEV_1_ %pred: 0.286  Ai and FEV_1_/FVC: 0.237  Ao and FEV_1_ %pred: 0.149  Ao and FEV_1_/FVC: 0.128  WA and FEV_1_ %pred: -0.007  WA and FEV_1_/FVC: -0.001 | NA |
| Lee et al, 2008[22]* | %LAA-950, MLD, Ai, WA, WA% | %LAA-950 and FEV_1_ %pred: -0.547  MLD and FEV_1_ %pred: 0.439  WA% and FEV_1_ %pred: -0.044 | %LAA-950 and FEV_1_ %pred: -0.553  MLD and FEV_1_ %pred: 0.619 |
| Lee et al, 2016* | ATI,E/I,E/I of MLD,Exp-856 | ATI and FEV_1_%Pred:-0.698,  ATI and FEV_1_/FVC:-0.709,  E/I of MLD and FEV_1_% Pred:-0.712,  E/I of MLD and FEV_1_/FVC: -0.714 | %LAA-856 and FEV_1_%Pred: -0.612,%LAA-856 and FEV_1_/FVC:-0.64 |
| Li Yan et al, 2020* | LAA%-950 | LAA% and FEV_1_%pred : -0.477  LAA% and FEV_1_ /FVC: -0.641 | NA |
| Li Yan et al, 2020* | LAA%-950ins,MLD | LAA%-950 and FEV_1_%pred :-0.400  LAA%-950 and FEV_1_ /FVC:-0.496 | NA |
| MacNeil et al, 2020* | LAA% | LAA% and FEV_1_ %pred :-0.64  LAA% and FEV_1_ /FVC:-0.83 | NA |
| Mochizuki et al, 2019* | LAA% | LAA% and FEV_1_:-0.34  LAA% and FEV_1_/FVC:-0.36  LAA% and FEV_25-75_:-0.47  LAA% and FEV_25-75_/FVC:-0.56 |  |
| Nishio et al, 2018 | LAV%,CSA%,WA% | LAV% and FEV_1_ :-0.505  LAV% and FEV_1_ /FVC:-0.640  CSA% and FEV_1_ :0.384  CSA% and FEV_1_ /FVC:0.288  WA% and FEV_1_ :-0.196  WA% and FEV_1_ /FVC:-0.131 | NA |
| Nishio et al, 2016* | %LAA,D | %LAA and FEV_1_:-0.565,  D and FEV_1_:0.0934 | NA |
| Occhipinti et al, 2019 | LAA%-950ins,LAA%-856exp,WT,fLDA%,pLDA% | LAA%-950 and FEV_1_%pred :-0.50  LAA%-950 and FEV_1_ /FVC:-0.67  WT and FEV_1_%pred :-0.22  WT and FEV_1_ /FVC:-0.11  fLDA% and FEV_1_%pred :-0.42  fLDA% and FEV_1_ /FVC:-0.49  pLDA% and FEV_1_%pred :-0.52  pLDA% and FEV_1_ /FVC:-0.68 | LAA%-856 and FEV_1_%pred :-0.58  LAA%-856 and FEV_1_ /FVC:-0.71 |
| Occhipinti et al, 2018* | %LAA-950,%LAA-856,%DLCO | %LAA-950 and FEV_1_/FVC: -0.66  %LAA-950 and %DLCO:-0.43 | %LAA-856 and FEV_1_/FVC : -0.64 ,%LAA-856 and %DLCO:-0.43 |
| Oh,S.Y et al, 2017* | %LAA-950 | %LAA-950 and FEV_1_%Pred: -0.533  %LAA-950 and FEV_1_/FVC: -0.663 | %LAA-950 and FEV_1_%Pred: -0.567 %LAA-950 and FEV_1_/FVC:-0.660 |
| Ohno et al, 2011 [30]* | WA%, etc. | WA% and FEV_1_ %pred: -0.69  WA% and FEV_1_/FVC: -0.59 | NA |
| Ohno et al, 2012* | FLA,%WA,WT | FLV and FEV_1_%Pred:0.64  FLV and FEV_1_/FVC:-0.58  %WA and FEV_1_% Pred:-0.69  %WA and FEV_1_/FVC: -0.59  WT and FEV_1_%Pred:-0.68  WT and FEV_1_/FVC:-0.62 | NA |
| Park et al, 2008 [23]* | %LAA-950 | %LAA-950 and FEV_1_ %pred: -0.46  %LAA-950 and FEV_1_/FVC: -0.67 | NA |
| Pauls et al, 2010 [24]* | %LAA-950, LV | %LAA-950 and FEV_1_ %pred: -0.360  LV and FEV_1_ %pred: -0.162 | NA |
| Saruya et al, 2016* | %LAA<-950,%CSA | %CSA and FEV_1_%Pred: 0.53,  LAA<-950 and FEV_1_% Pred:-0.41 | NA |
| Sasaki et al, 2014* | LA,%WA | LA and FEV_1_%Pred:0.443  LA and FEV_1_/FVC:0.512  %WA and FEV_1_% Pred:-0.499,  %WA and FEV_1_/FVC: -0.515 | NA |
| Sileikiene et al, 2017* | %LAV-950 | %LAV and FEV_1_%Pred:-0.505  %LAV and FEV_1_/FVC:-0.640 | No CT quantitative measurements |
| Suzuki et al, 2020* | LAA%,HAA%,AA% | LAA% and FEV_1_%pred :-0.29  LAA% and FEV_1_ /FVC:-0.23  HAA% and FEV_1_%pred :-0.29  HAA% and FEV_1_ /FVC:0.29  AA% and FEV_1_%pred :-0.45  AA% and FEV_1_ /FVC:0.13 | NA |
| Timmins et al, 2012* | %LAA<910 | %LAA<910 and FEV_1_%Pred: -0.28  %LAA<910 and FEV_1_/FVC:-0.57 | NA |
| Wang et al, 2015* | %LAV-950 | %LAV-950 and FEV_1_% Pred: -0.67  %LAV-950 and FEV_1_/FVC:-0.68 | NA |
| Washko et al, 2009 [25]* | %LAA-950, WA%, Ai, WT | WA% and FEV_1_ %pred: -0.28  WA% and FEV_1_/FVC: -0.014  Ai and FEV_1_ %pred: 0.14  Ai and FEV_1_FVC: 0.07  WT and FEV_1_ %pred: -0.13  WT and FEV_1_/FVC: -0.05 | NA |
| Yahaba et al, 2014* | Ai,%WA | Ai and FEV_1_:0.50  %WA and FEV_1_:-0.53 | Ai and FEV_1_:0.34  %WA and FEV_1_:-0.40 |
| Yamashiro et al, 2010 [26]* | %LAA-950, MLD, LV, etc. | %LAA-950 and FEV_1_ %pred: -0.625  %LAA-950 and FEV_1_/FVC: -0.713  MLD and FEV_1_.%pred: 0.494  MLD and FEV_1_/FVC: 0.562  LV and FEV_1_ %pred: -0.010  LV and FEV_1_/FVC: -0.198 | %LAA-950 and FEV_1_ %pred: -0.637  %LAA-950 and FEV_1_/FVC: -0.729  MLD and FEV_1_.%pred: 0.661  MLD and FEV_1_/FVC: 0.743  LV and FEV_1_ %pred: -0.406  LV and FEV_1_/FVC: -0.588 |
| Yamashiro et al, 2010 [28]* | %LAA-950, WA%, Ai, etc. | %LAA-950 and FEV_1_ %pred: -0.460  WA% and FEV_1_ %pred: -0.470  Ai and FEV_1_ %pred: 0.450 | NA |
| Zhang et al, 2018* | LAA%-950,P15-_IN_,P15-_EX_,E/I _MLD_ | LAA% and FEV_1_ %pred :0.10  LAA% and FEV_1_ /FVC:-0.21  LAA% and RV/TLC:0.59  LAA% and DLCO/VA:-0.62  P15-_IN_ and FEV_1_ %pred :-0.29  P15-_IN_ and FEV_1_ /FVC :0.26  P15-_IN_ and RV/TLC:-0.40  P15-_IN_ and DLCO/VA:0.53  E/I _MLD_ and FEV_1_ %pred :-0.00  E/I _MLD_ and FEV_1_ /FVC :-0.19  E/I _MLD_ and RV/TLC:0.06  E/I _MLD_ and DLCO/VA:-0.19 | P15-_EX_ and FEV_1_ %pred :-0.22  P15-_EX_ and FEV_1_ /FVCpred :0.33  P15-_EX_ and RV/TLC:-0.23  P15-_EX_ and DLCO/VA:0.55 |
| Zhang et al, 2008 [27]* | %-LAA-950, -910, MLD | %LAA-950 and FEV_1_ %pred: -0.520  %LAA-950 and FEV_1_/FVC: -0.626  MLD and FEV_1_ %pred: 0.416  MLD and FEV_1_/FVC: 0.512  %LAA-910 and FEV_1_ %pred: -0.437  %LAA-910 and FEV_1_/FVC: -0.548 | NA |
| Zhang et al, 2015* | %LAA-950,%WA | %LAA-950 and FEV_1_%Pred: -0.65  %LAA-950 and FEV_1_/FVC : -0.6  %WA and FEV_1_% Pred: -0.54  %WA and FEV_1_/FVC: -0.45 | NA |
| Zhao et al, 2019* | LAA%-950ins,LAA%-856exp,Emph% | LAA%-950 and FEV_1_%pred :-0.272  LAA%-950 and FEV_1_ /FVC:-0.458  LAA%-950 and DLCO:-0.347  LAA%-950 and RV/TLC:0.230  Emph% and FEV_1_ /FVC:-0.358  Emph% and DLCO:=-0.422  Emph% and RV/TLC:0.342 | LAA%-856 and FEV_1_%pred :-0.210  LAA%-856 and FEV_1_ /FVC:-0.276  LAA%-856 and DLCO:0.222  LAA%-856 and RV/TLC:0.159 |

NA = Not available; %LAA = Percentage low attenuation area; MLD = Mean lung density; LV = Lung volume; Perc = Percentile point of lung density; WA% = Airway wall area percentage; Ai = Airway lumen area; Ao = Total airway area; WT = Wall thickness; T/D radio = Ratio of airway wall thickness to total diameter.

* Included in the meta-analysis.

† Expressed as *r^2^*.
