# Supplementary material for: Quantitative CT parameters correlate with lung function in chronic obstructive pulmonary disease: A systematic review and meta-analysis
Source: Front Surg. 2023 Jan 4;9:1066031. doi: 10.3389/fsurg.2022.1066031 (PMC9845891; doi:10.3389/fsurg.2022.1066031)
Supplement: Supplementary Table S6 — Assessment of Study Quality, by the Quality Assessment of Diagnostic Accuracy Studies (QUADAS) tool [file Table7.docx]

**Electronic supplementary table 7 Assessment of Study Quality, by the Quality Assessment of Diagnostic Accuracy Studies (QUADAS) tool**

| **Study, Year (Reference)** | **Item1: Representative patient sample** | **Item2: Selection criteria clearly described** | **Item3: Acceptable reference standard*** | **Item4: Acceptable delay between tests^†^** | **Item5: Partial verification avoided** | **Item6: Differential verification avoided** | **Item7: Incorporation avoided** | **Item8: Adequate index test description*** | **Item9:Adequate reference standard description*** | **Item10: Index test blinded to reference standard*** | **Item11: Reference standard blinded to index test*** | **Item12: Clinical data available as in practice** | **Item13: Uninterpretable test results reported** | **Item14: Withdrawals explained** | **Score^†^** |
| --- | --- | --- | --- | --- | --- | --- | --- | --- | --- | --- | --- | --- | --- | --- | --- |
| Akira et al, 2009 [13] | Y | Y | Y | Y | Y | Y | Y | Y | Y | U | U | Y | Y | Y | 13.0 |
| Bon et al, 2009 [18] | Y | Y | Y | U | Y | Y | Y | Y | Y | U | Y | Y | Y | Y | 13.0 |
| Dransfield et al, 2007 [19] | Y | Y | Y | U | Y | Y | Y | Y | Y | U | U | Y | Y | Y | 12.5 |
| Hasegawa et al, 2006 [20] | Y | Y | Y | Y | Y | Y | Y | Y | Y | Y | U | Y | Y | Y | 13.5 |
| Hesselbacher et al, 2011 [29] | Y | Y | Y | U | Y | Y | Y | Y | Y | U | Y | Y | Y | Y | 13.0 |
| Iwasawa et al, 2011 [31] | Y | Y | Y | Y | Y | Y | Y | Y | Y | Y | U | Y | Y | Y | 13.5 |
| Leader et al, 2008 [21] | Y | Y | Y | U | Y | Y | Y | Y | Y | U | U | Y | Y | Y | 12.5 |
| Lee et al, 2008 [22] | Y | Y | Y | Y | Y | Y | Y | Y | Y | Y | U | Y | Y | Y | 13.5 |
| Ohno et al, 2011 [30] | Y | Y | Y | Y | Y | Y | Y | Y | Y | U | U | Y | Y | Y | 13.0 |
| Park et al, 2008 [23] | Y | Y | Y | Y | Y | Y | Y | Y | Y | U | U | Y | Y | Y | 13.0 |
| Pauls et al, 2010 [24] | Y | Y | Y | Y | Y | Y | Y | Y | Y | Y | U | Y | Y | Y | 13.5 |
| Washko et al, 2009 [25] | Y | Y | Y | U | Y | Y | Y | Y | Y | Y | U | Y | Y | Y | 13.0 |
| Yamashiro et al, 2010 [26] | Y | Y | Y | U | Y | Y | Y | Y | Y | U | U | Y | Y | Y | 12.5 |
| Yamashiro et al, 2010 [28] | Y | Y | Y | U | Y | Y | Y | Y | Y | Y | U | Y | Y | Y | 13.0 |
| Zhang et al, 2008 [27] | Y | Y | Y | Y | Y | Y | Y | Y | Y | U | U | Y | Y | Y | 13.0 |
| Capaldi et al, 2016* | Y | Y | Y | U | Y | Y | Y | Y | Y | U | U | Y | Y | Y | 12.5 |
| Gawlitza et al, 2018* | Y | Y | Y | U | Y | Y | Y | Y | Y | U | Y | Y | Y | Y | 13.0 |
| Haraguchi et al, 2016* | Y | Y | Y | U | Y | Y | Y | Y | Y | Y | U | Y | Y | Y | 13.0 |
| Hoshino et al, 2014* | Y | Y | Y | Y | Y | Y | Y | Y | Y | Y | U | Y | Y | Y | 13.5 |
| Ju et al, 2014* | Y | Y | Y | U | Y | Y | Y | Y | Y | U | Y | Y | Y | Y | 13.0 |
| Karayama et al, 2017* | Y | Y | Y | Y | Y | Y | Y | Y | Y | Y | U | Y | Y | Y | 13.5 |
| Kim et al, 2013* | Y | Y | Y | U | Y | Y | Y | Y | Y | U | U | Y | Y | Y | 12.5 |
| Kim et al, 2015* | Y | Y | Y | U | Y | Y | Y | Y | Y | Y | U | Y | Y | Y | 13.0 |
| Kundu et al, 2013* | Y | Y | Y | Y | Y | Y | Y | Y | Y | U | U | Y | Y | Y | 13.0 |
| Kurashima et al, 2013* | Y | Y | Y | Y | Y | Y | Y | Y | Y | U | U | Y | Y | Y | 13.0 |
| Lee et al, 2016* | Y | Y | Y | Y | Y | Y | Y | Y | Y | Y | U | Y | Y | Y | 13.5 |
| Nishio et al, 2016* | Y | Y | Y | U | Y | Y | Y | Y | Y | Y | U | Y | Y | Y | 13.0 |
| Occhipinti et al, 2018* | Y | Y | Y | U | Y | Y | Y | Y | Y | U | U | Y | Y | Y | 12.5 |
| Oh,S.Y et al, 2017* | Y | Y | Y | U | Y | Y | Y | Y | Y | Y | U | Y | Y | Y | 13.0 |
| Ohno et al, 2012* | Y | Y | Y | Y | Y | Y | Y | Y | Y | U | U | Y | Y | Y | 13.0 |
| Saruya et al, 2016* | Y | Y | Y | Y | Y | Y | Y | Y | Y | U | U | Y | Y | Y | 13.0 |
| Sasaki et al, 2014* | Y | Y | Y | U | Y | Y | Y | Y | Y | U | Y | Y | Y | Y | 13.0 |
| Sileikiene et al, 2017* | Y | Y | Y | U | Y | Y | Y | Y | Y | U | U | Y | Y | Y | 12.5 |
| Timmins et al, 2012* | Y | Y | Y | Y | Y | Y | Y | Y | Y | Y | U | Y | Y | Y | 13.5 |
| Wang et al, 2015* | Y | Y | Y | U | Y | Y | Y | Y | Y | U | Y | Y | Y | Y | 13.0 |
| Yahaba et al, 2014* | Y | Y | Y | Y | Y | Y | Y | Y | Y | U | U | Y | Y | Y | 13.0 |
| Zhang et al, 2015* | Y | Y | Y | U | Y | Y | Y | Y | Y | U | U | Y | Y | Y | 12.5 |
| Item number (Yes) | 15 | 15 | 15 | 8 | 15 | 15 | 15 | 15 | 15 | 6 | 2 | 15 | 15 | 15 |  |
| Item number (No) | 0 | 0 | 0 | 0 | 0 | 0 | 0 | 0 | 0 | 0 | 0 | 0 | 0 | 0 |  |
| Item number (Unknown) | 0 | 0 | 0 | 7 | 0 | 0 | 0 | 0 | 0 | 9 | 13 | 0 | 0 | 0 |  |

Y = Yes; U = Unclear; N = No.

* CT qualification was considered as index test in item 8, 10, 11; PFT was considered as reference standard in item 3, 9, 10, 11.

† Maximum delay of 30 days between CT and PFT was considered as acceptable.
