# Supplementary material for: Quantitative CT parameters correlate with lung function in chronic obstructive pulmonary disease: A systematic review and meta-analysis
Source: Front Surg. 2023 Jan 4;9:1066031. doi: 10.3389/fsurg.2022.1066031 (PMC9845891; doi:10.3389/fsurg.2022.1066031)
Supplement: Supplementary Table S7 — Bias analysis in the meta-analysis [file Table8.docx]

**Electronic supplementary table 8 Bias analysis in the meta-analysis**

|  | **Heterogeneity** | | **Publication bias** | | **Meta-regression for gender*** |
| --- | --- | --- | --- | --- | --- |
|  | ***P*** | **I^2^ ,%** | **Begg and Mazumdar rank correlation, *P*** | **Egger’s regression, *P*** | ***P*** |
| **Inspiration** |  |  |  |  |  |
| %LAA-950 and FEV_1_ %pred | 0.001 | 49.3% | 0.62 | 0.22 | 0.16 |
| %LAA-950 and FEV_1_/FVC | 0.017 | 41.5% | 0.45 | 0.31 | 0.71 |
| %LAA＜-950 and FEV_1_ %pred | 0.499 | 0 | 0.37 | 0.28 | 0.21 |
| %LAA＜-950 and FEV_1_/FVC | 0.492 | 0 | 0.42 | 0.34 | 0.77 |
| MLD and FEV_1_ %pred | 0.044 | 48.1% | 0.50 | 0.14 | 0.34 |
| MLD and FEV_1_/FVC | 0.151 | 33.4% | 0.60 | 0.77 | 0.71 |
| WA% and FEV_1_ %pred | 0.031 | 52.8% | 0.73 | 0.60 | 0.64 |
| WA% and FEV_1_/FVC | 0.345 | 11.1% | 0.61 | 0.11 | 0.11 |
| Perc15 and FEV_1_%pred | 0.696 | 0 | 0.36 | 0.17 | 0.32 |
| Perc15 and FEV_1_/FVC | 0.499 | 0 | 0.57 | 0.16 | 0.46 |
| WT and FEV_1_ %pred | 0 | **96.1%** | 0.21 | 0.32 | IS |
| WT and FEV_1_/FVC | 0 | **95%** | 0.08 | 0.37 | IS |
| ATI and FEV_1_ %pred | 0 | **96.8%** | 0.53 | 0.44 | 0.22 |
| ATI and FEV_1_/FVC | 0.225 | 32.9% | 0.24 | 0.47 | 0.06 |
| AI and FEV_1_ %pred | 0.020 | 65.9% | 0.73 | 0.62 | IS |
| AI and FEV_1_/FVC | 0.066 | 58.4% |  |  |  |
| **Expiration** |  |  |  |  |  |
| %LAA-950 and FEV_1_ %pred | 0.354 | 9.8% | 0.12 | 0.21 | 0.85 |
| %LAA-950 and FEV_1_/FVC | 0.787 | 0 | 0.32 | 0.66 | IS |
| %LAA＜-950 and FEV_1_ %pred | 0.069 | 44.9% | 0.69 | 0.32 | 0.23 |
| %LAA＜-950 and FEV_1_/FVC | 0.423 | 1.7% | 0.31 | 0.47 | 0.54 |
| MLD and FEV_1_ %pred | 0.231 | 28.6% | 0.12 | 0.15 | 0.38 |
| MLD and FEV_1_/FVC | 0.396 | 1.9% | 0.42 | 0.17 | 0.17 |
| **The brand of CT machine** |  |  |  |  |  |
| %LAA-950 and FEV_1_ %pred(GE） | 0.129 | 37.7% | 0.08 | 0.34 | 0.56 |
| %LAA-950 and FEV_1_ %pred（non-GE)） | 0.262 | 21% | 0.27 | 0.69 | IS |
| **Radiation Dose** |  |  |  |  |  |
| Normal | 0.023 | 42.7% | 0.39 | 0.07 | 0.66 |
| Low | 0.007 | 53.6% | 0.31 | 0.36 | 0.29 |

FEV_1_ %pred = Predicted forced expiratory volume in the first second; FEV_1_/FVC = FEV_1_ divided by forced vital capacity; %LAA-950 = Percentage lower attenuation area than -950HU; MLD = Mean lung density; Perc15 = 15 percentile point of lung density; WA% = Wall area percentage; IS = Insufficient study numbers to perform analysis.

*: Percentage of male was considered as moderator variable.
